# The dose-effect relationship of acupuncture on limb dysfunction after acute stroke: a systematic review and meta-analysis

**DOI:** 10.3389/fneur.2024.1341560

**Published:** 2024-02-28

**Authors:** Xiaoxuan Wang, Lu Xiao, Lingyong Xiao, Chunlei Tian, Yi Liu, Xiaoyu Dai

**Affiliations:** ^1^Department of Acupuncture and Moxibustion, The First Affiliated Hospital of Tianjin University of Traditional Chinese Medicine, Tianjin, China; ^2^National Clinical Research Center of Acupuncture and Moxibustion of Traditional Chinese Medicine, Tianjin, China; ^3^Department of Emergency, The First Affiliated Hospital of Tianjin University of Traditional Chinese Medicine, Tianjin, China

**Keywords:** acupuncture, dose-related, acute stroke, limb dysfunction, MCID

## Abstract

**Objective:**

This study aimed to investigate the relationship between the dose and efficacy of acupuncture in treating limb dysfunction during acute stroke.

**Methods:**

Studies were searched from seven databases, including PubMed, Embase, Cochrane Library, Chinese National Knowledge Infrastructure (CNKI), Wanfang Data (WF), VIP information database (VIP), and China Biology Medicine Database (CBM). All databases were searched until August 1, 2023 from inception. The risk of bias was assessed using Cochrane Collaboration’s risk of bias tool (RoB2). Meta-analyses were performed using RevMan V.5.4 and Stata 12.0 statistical software. We used Fugl-Meyer Assessment (FMA) to measure recovery of limb dysfunction, NIH Stroke Scale (NIHSS) to measure neurological deficits, and Barthel index, Modified Barthel Index (MBI), and Activities of Daily Living (ADL) to measure activities of daily living. The primary outcome measure is FMA. After examining and integrating the raw data, we performed a meta-analysis using a 3-step process. First, we investigated the dose-related effects of acupuncture at varying doses and determined the optimal dosage for maximum therapeutic benefits. Second, we determined the difference between post-intervention and baseline scores on the outcomes of interest to determine minimal clinically important differences (MCID) to provide evidence for clinical treatment. Third, by combining the results of step 1 and step 2, we made the recommendations employing the Grades of Recommendations, Assessment, Development and Evaluations (GRADE) tool.

**Results:**

Twenty-six studies containing 1947 participants were included, among which 61.5% of RCTs had a low risk of bias. Through the three-step analysis, the effect in improving limb dysfunction of acute stroke varied across different acupuncture dosages. Regarding the frequency of acupuncture, the results demonstrated a significant improvement in the low (every other day) and moderate-frequency (once a day) groups (low frequency: MD: 9.02, 95%CI: 5.40–12.64, *p* < 0.00001; moderate frequency: MD: 10.11, 95%CI: 5.05–15.18, *p* < 0.00001, heterogeneity (*p* = 0.87), *I*^2^ = 0%). For the acupuncture retention time, the results showed no significant difference between the short and medium retention groups (short retention time: MD: 0.05, 95% CI: −0.21–0.31, *p* = 0.71; medium retention time: MD: −1.16, 95% CI: −2.80–0.48, *p* = 0.17, heterogeneity (*p* < 0.00001), *I*^2^ = 99%). For the course of acupuncture, the results showed a significant improvement in the short course treatment (less than 2 weeks) group (MD: 14.87, 95% CI: 12.18–17.56, *p* < 0.00001, heterogeneity (*p* = 0.45), *I*^2^ = 0%).

**Conclusion:**

Our study demonstrated the effectiveness of different acupuncture dose in improving limb dysfunction. The pooled data suggested that the optimal intervention dose for acupuncture interval time was low (every other day) and moderate frequency (once a day), the optimal intervention dose for needle course time was short course treatment (less than 2 weeks). But we did not find the optimal intervention dose for needle retention time. Future studies of higher quality are needed to confirm this.

**Systematic Review Registration:**https://www.crd.york.ac.uk/PROSPERO/, CRD42023447202.

## 1 Introduction

Stroke has become one of the leading causes of death and disability worldwide, and its incidence is increasing yearly (1). Fifteen million people die from stroke each year worldwide (2), with up to 85% of survivors experiencing various degrees of physical dysfunction shortly after the stroke (3) and approximately 40% being severely disabled. The main clinical manifestations were partial or complete impairment of daily living activities, reducing the individual’s quality of life (4). And few people fully recover after 6 months of stroke (5).

Stroke may result in extensive biochemical, anatomical, and physiological alterations that can affect brain function. The brain relies on neuroplasticity within residual neural circuits to regain lost function caused by injury (6). Current knowledge regarding brain repair processes indicates that the most substantial recovery typically transpires during the initial weeks following a stroke (7). Therefore, timely and effective interventions for neurorehabilitation are essential for the recovery of limb dysfunction. Treatments for limb dysfunction include western medicine (WM), rehabilitation training, and acupuncture in China (8). Acupuncture has been widely used in treating limb dysfunction caused by stroke because of its safety and effectiveness (9). Meta-analysis indicated that acupuncture was effective in improving limb dysfunction after acute stroke, but there was heterogeneity among literature (10, 11). Acupuncture dose was an important cause for heterogeneity (12). The acupuncture dosage which is closely related to the therapeutic effect is a multifaceted matter that encompasses several crucial factors, including but not limited to the frequency, the needle retention time, and the overall treatment course. To date, there is a paucity of meta-analysis that specifically examines the optimal dosage of acupuncture.

Therefore, the study aims to evaluate the efficacy of acupuncture in treating limb dysfunction for acute stroke patients. It seeks to investigate the dose-effect relationship of acupuncture in treating limb dysfunction of acute stroke, identify the optimal acupuncture protocol, and provide evidence for the clinical treatment of limb dysfunction for acute stroke patients.

## 2 Methods

### 2.1 Defining the dose components of acupuncture treatment

We have chosen these acupuncture dosage based on the theory from ancient medical books and results from relevant meta-analysis. Firstly, acupuncture is one of the traditional treatment methods of Chinese medicine. As early as in the classical medical book “Huangdi Neijing,” there are relevant records about acupuncture dosage. It is believed that the interval time of acupuncture, retention time of acupuncture and course of treatment are closely related to the curative effect. Nowadays, Prof Shi Xuemin, an outstanding and famous doctor of acupuncture in China, put forward the concept of acupuncture dose, believing that acupuncture dose consist of the interval time of acupuncture, retention time of acupuncture and course of treatment. Secondly, there are also meta-analyses and reviews that indicate acupuncture dose should include the number of needled points, de qi response, frequency and course of treatment. In our review, the number of needled points and de qi response were not selected, but the retention time of acupuncture was added. Because we believe that the effectiveness of acupuncture is closely associated with combination of acupoints according to theories of traditional Chinese medicine, such as the method of Shu-mu points combination, Yuan-luo acupoints combined from “Huangdi Neijing,” which is less relevant to with the number of acupuncture points. De qi is the premise of efficacy of acupuncture, acupuncture without de qi is nonstandard, cannot be called acupuncture treatment. Therefore, the acupuncture dose selected by us includes the interval time of acupuncture, acupuncture retention time, and the course of treatment.

### 2.2 Searching strategy

This systematic review was developed based on the Preferred Reporting Items for Systematic reviews and Meta-Analyses (PRISMA) and checked by the PRISMA checklist (PRISMA checklist is provided in the Supplementary Table S1). The protocol of the study was registered on PROSPERO (CRD42023447202).

We searched the following databases from inception to August 1, 2023: PubMed, Embase, Cochrane Library, Chinese National Knowledge Infrastructure (CNKI), Wanfang Data (WF), VIP information database (VIP), and China Biology Medicine Database (CBM). We also searched the reference lists of relevant reports and review articles identified from electronic databases for potential eligible studies. The language was limited to English and Chinese. The keywords used for the search consist of four parts: acupuncture (e.g., needle), acute (e.g., early), stroke (e.g., cerebrovascular disease), limb dysfunction (e.g., limb motor impairment). Search strategy for PubMed is presented in Table 1.

**Table 1:** Search strategy for PubMed.

	Search strategy
#1	Search: “acupuncture”[MeSH Terms]
#2	Search: (“needling” [Title/Abstract] OR “needle” [Title/Abstract] OR “needle puncturing” [Title/Abstract] OR “needle puncture” [Title/Abstract] OR “needling method” [Title/Abstract] OR “acupuncture” [Title/Abstract] OR “moxibustion” [Title/Abstract] OR “head acupuncture” [Title/Abstract] OR “scalp needl*” [Title/Abstract] OR “hand acupuncture” [Title/Abstract] OR “manual acupuncture” [Title/Abstract] OR “hand needle” [Title/Abstract] OR “body acupuncture” [Title/Abstract] OR “wrist ankle acupuncture” [Title/Abstract] OR “wrist ankle needle” [Title/Abstract])
#3	Search: (“acute” [Title/Abstract] OR “acute stage” [Title/Abstract] OR “early” [Title/Abstract])
#4	Search: “stroke” [MeSH Terms]
#5	Search: (“cerebral ischemia” [Title/Abstract] OR “brain ischemia” [Title/Abstract] OR “cerebral infarction”[Title/Abstract] OR “brain infarction” [Title/Abstract] OR “cerebral embolism” [Title/Abstract] OR “brain embolism” [Title/Abstract] OR “cerebral thrombosis”[Title/Abstract] OR “cerebral thrombosis” [Title/Abstract] OR “cerebrovascular accident” [Title/Abstract] OR “brain vascular accident” [Title/Abstract] OR “cerebrovascular disease” [Title/Abstract])
#6	Search: (“limb dysfunction” [Title/Abstract] OR “limb motor impairment” [Title/Abstract] OR “limb dyskinesia” [Title/Abstract] OR “limb motor dysfunction” [Title/Abstract] OR “limb movement disorder” [Title/Abstract] OR “paralyzed” [Title/Abstract] OR “hemiplegia” [Title/Abstract])
#7	#1 OR #2
#8	#4 OR #5
#9	#7 AND #3 AND #8 AND #5

### 2.3 Eligibility criteria

The inclusion criteria were as follows: (1) Type of included studies: only RCTs of acupuncture therapy for limb dysfunction during acute stroke were included. (2) Participants: the participants were diagnosed with acute stroke based on CT or MRI, which revealed either cerebral infarction. Additionally, they exhibited limb dysfunction. (3) Intervention and comparison: the treatment group was treated with hand acupuncture combined with the conventional western treatment while the control group was treated with the conventional western treatments or placebo. (4) Outcomes measures: The primary outcome was Fugl-Meyer Assessment (FMA). The secondary outcomes were NIH Stroke Scale (NIHSS), Barthel index (BI), Modified Barthel Index (MBI), Activities of Daily Living (ADL).

The exclusion criteria were as follows: (1) Animal studies, non-RCTs, such as reviews, case series, conference proceedings. (2) RCTs used electroacupuncture, auricular acupuncture or eye acupuncture, and other special acupuncture methods. (3) Studies did not use relevant scales as outcome. (4) Duplicate studies.

### 2.4 Selection of studies

Two authors (WX and XL) independently screened the study title and abstract and excluded the studies that obviously did not meet the criteria. Then full text will be read carefully to determine the final studies to be included. Any discrepancies were resolved through further discussion with the third author (XY).

### 2.5 Data extraction

Two authors (WX and XL) independently conducted data extraction using a predefined form. The following items were extracted: title, author, publication year, sample size, age, gender, random method, intervention measures for experimental group and control group, acupuncture manipulation, interval time, retention time, course of treatment, outcome measures, and adverse events. Where data were unobtainable, attempts were made to acquire them through Email or direct communication with the corresponding author of the pertinent article.

### 2.6 Risk of bias

Two authors (WX and XL) assessed the quality of eligible trials by the Cochrane Collaboration’s risk of bias tool (RoB2) (13). The overall quality was rated in 3 categories: low risk of bias, some concerns, or high risk of bias according to the following items: randomization process, deviations from intended interventions, measurement of the outcome, and selection of the reported result. For a study to be rated at overall low risk of bias, all of the domains needed to be rated at low risk of bias. Any discrepancies during the evaluation process were discussed with the third reviewer (XY) for the solution.

### 2.7 Quality assessment

The Grades of Recommendations, Assessment, Development and Evaluations (GRADE) tool was used to assess the quality of the certainty of evidence (14) of the outcomes independently by two authors (WX and XL). The quality was classified into high, moderate, low or very low, and five reasons to down the quality of each evidence are as follows: (1) study design; (2) rise of bias (3) inconsistency of results; (4) evidence of indirectness; (5) imprecision. Disagreements on the quality of evidence were resolved by a third reviewer.

### 2.8 Statistical analysis

Data were analyzed using RevMan 5.4 and Stata 12.0 software. Dichotomous data were presented as relative risks (RR). Continuous data were presented as mean differences (MD). Each effect size was expressed as a 95% confidence interval (CI). Heterogeneity was checked by the *I^2^* statistic with significant heterogeneity defined as the *I^2^* > 50. A fixed effects model was chosen for no significant heterogeneity; otherwise, random effects were selected. If quantitative analysis was not feasible, it was recommended to employ descriptive analysis methods. To explore the most suitable or optimal parameters of acupuncture dose, the included studies were divided into different groups. According to the acupuncture interval time, they were divided into three groups: low-frequency (every other day), moderate-frequency (once a day), and high-frequency (twice a day). According to the acupuncture retention time, they were divided into three groups: short retention time (≤20 min), medium retention time (20–30 min), and long retention time (more than 30 min). According to the course of treatment, they were divided into three groups: short course treatment (less than 2 weeks), medium course treatment (2–4 weeks), and long course treatment (more than 4 weeks).

Sensitivity analyses were assessed by removing any single study in each group to explore its effect on the heterogeneity. Publication bias was presented by the funnel plots and Egger’s test.

The minimum clinically important difference (MCID) was defined as the minimal magnitude an subjective outcome must change to gain clinical effectiveness meeting the satisfaction of patients or clinicians (15). The value of MCID could be obtained based on previous researches (16). Therefore, we searched the relevant literature and gained the value of MCID. Accepted MCID scores were applied to evaluate efficacy: FMA > 6 points (17), BI>1.85 points (18), MBI > 5.34 points (19), ADL > 5.9 points (20). We did not evaluate the NIHSS scale because there were no studies to illustrate the MCID value of the NIHSS scale.

## 3 Results

**Figure 1:**
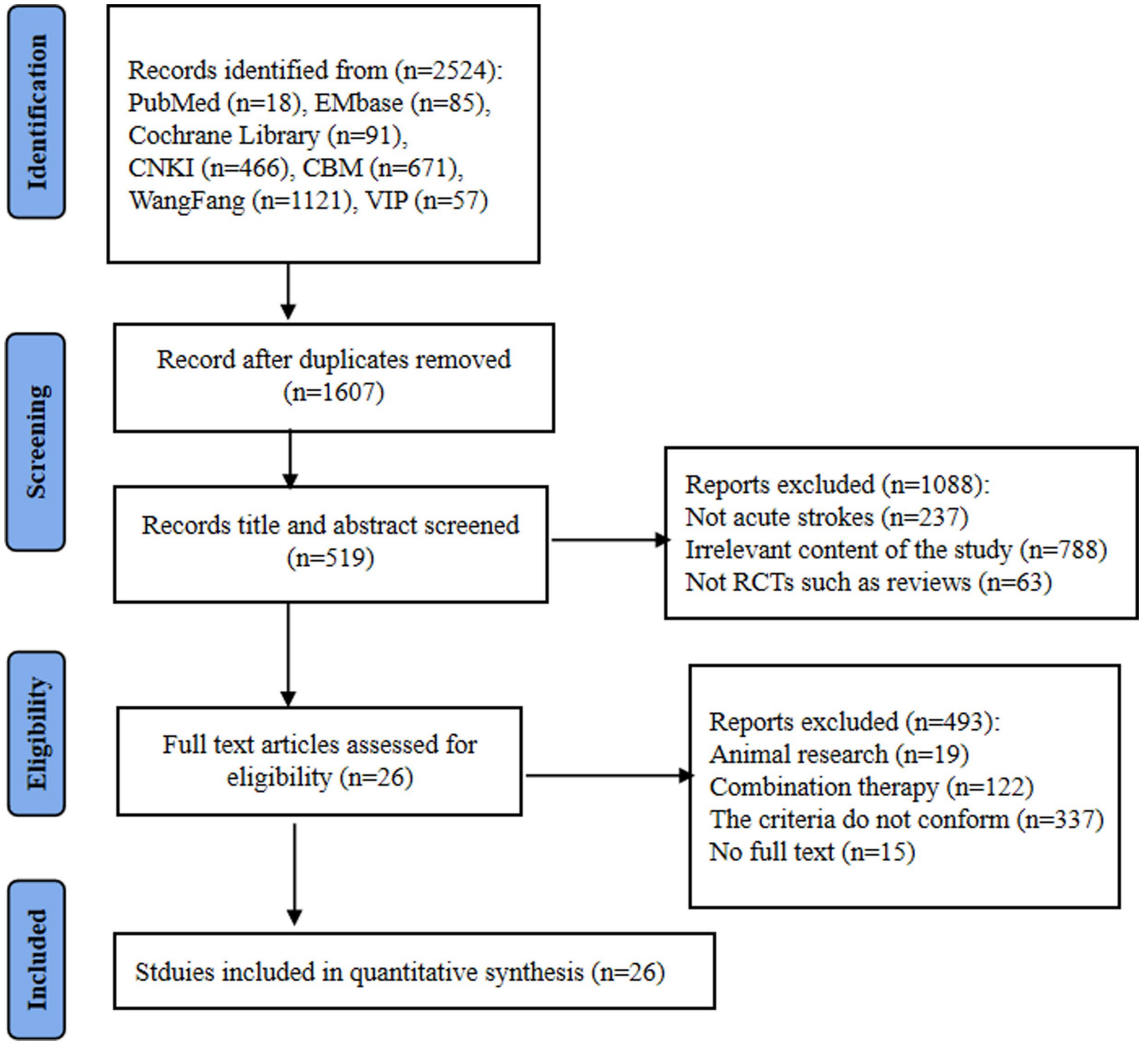
Flowchart of the study selection.

A total of 26 studies were finally included, consisting of 22 Chinese and 4 English studies. The studies search yielded 1947 potentially relevant records, 917 were duplicated studies and were excluded. After screening titles and abstracts of the remaining 1,607 records, 1,088 records were excluded for the following reasons: (1) not acute strokes (*n* = 237), (2) irrelevant content, such as not limb dysfunction (*n* = 788) (3) not RCTs, such as reviews (*n* = 63). By browsing the full text of the remaining 519 records, 493 studies were excluded for the following reasons: (1) the criteria was not satisfied (68%), (2) combination therapy (25%), (3) animal research and no full text (7%). A flowchart of study is presented in Figure 1.

### 3.1 Study characteristics

A total of 26 studies (1947 participants) were included. Among these studies, 25 studies compared combination therapy of routine medicine and acupuncture with routine medicine (e.g., WM, rehabilitation etc.) alone one study compared combination therapy of routine medicine and acupuncture with combination therapy of routine medicine and sham acupuncture. Sample sizes ranged from 6 to 60 in the experimental and control groups, with mean (SD) age ranging from 44.35 (11.02) years old to 73.67 (3.48) years old.

Seventeen studies used FMA (21–37) to evaluate the limb dysfunction improvement, sixteen studies used NIHSS (21, 25–27, 29–31, 36, 38–44) to evaluate the neurological deficits. Seventeen studies used BI (23–27, 29–31, 33–35, 37, 38, 42–45), MBI (21, 46) mentioned in only two studies, and ADL (36, 39, 40, 46) mentioned in four studies to evaluate the activities of daily living. The study characteristics are shown in Table 2.

**Table 2:** Characteristics of included studies.

Study	Sample size (T/C)	Age (mean ± sd)	Treatment group	Control group	Retaining time	Frequency	Course	Outcome
Wei et al. (15)	20/20	T: 53.17 ± 6.15 C: 51.09 ± 7.22	RM+ acupuncture	RM	30 min	Per day	28 days	① + ② + ④
Ma et al. (16)	–	T: 68.79 ± 6.43 C: 68.43 ± 6.01	RM+ acupuncture	RM	30 min	Per day	14 days	② + ③
Sheng et al. (17)	50/40	T: 59.71 ± 17.29 C: 60.15 ± 17.85	RM+ acupuncture	RM	30 min	Per day	14 days	④ + ⑤
Chen et al. (18)	30/30	T: 62.59 ± 5.29 C: 61.80 ± 5.38	RM+ acupuncture	RM	30 min	Per day	8 days	② + ⑤
Fan et al. (19)	51/51	T: 72.53 ± 3.25 C: 73.67 ± 3.48	RM+ acupuncture	RM	20 min	Per day	28 days	①
Zhang et al. (20)	43/43	T: 61.48 ± 5.18 C: 60.79 ± 5.27	RM+ acupuncture	RM	30 min	Per day	8 days	② + ⑤
Wang et al. (21)	42/41	T: 65.03 ± 4.64 C: 64.93 ± 4.61	RM+ acupuncture	RM	30 min	Every other day	30 days	① + ③
Liu et al. (38)	6/7	T: 59.67 ± 2.50 C: 64.43 ± 7.37	RM+ acupuncture	RM	30 min	Twice a day	6 days	②
Wu et al. (46)	43/43	T: 65.4 ± 5.7 C: 65.7 ± 5.8	RM+ acupuncture	RM	30 min	Per day	14 days	① + ③
Li et al. (39)	55/55	T: 59.60 ± 6.82 C: 61.2 ± 8.68	RM+ acupuncture	RM	30 min	Per day	14 days	① + ② + ③
Wang et al. (22)	60/60	T: 62.4 ± 9.0 C: 59.5 ± 8.9	RM+ acupuncture	RM	30 min	Per day	14 days	① + ② + ③
Du et al. (40)	32/32	T: 44.35 ± 11.02 C: 45.26 ± 12.07	RM+ acupuncture	RM	30 min	Per day	28 days	② + ③
Su et al. (23)	40/40	T: 68.20 ± 5.5 C: 67.94 ± 5.5	RM+ acupuncture	RM	30 min	Per day	14 days	① + ② + ③
Cheng et al. (41)	50/50	T: 64.2 ± 4.7 C: 64.4 ± 4.9	RM+ acupuncture	RM	30 min	Per day	28 days	①
Liao et al. (24)	28/20	T: 62.29 ± 12.33 C: 55.45 ± 15.22	RM+ acupuncture	RM + sham acupuncture	20 min	Every other day	56 days	② + ③
Chu et al. (25)	60/60	T: 71 ± 8 C: 69 ± 8	RM+ acupuncture	RM	30 min	Per day	14 days	① + ② + ③
Tian et al. (26)	58/57	T: 62.45 ± 9.02 C: 59.55 ± 8.88	RM+ acupuncture	RM	30 min	Per day	14 days	① + ② + ③
Wu et al. (42)	48/48	T: 58.60 ± 3.10 C: 61.30 ± 4.20	RM+ acupuncture	RM	30 min	Per day	45 days	② + ③
Liu et al. (27)	18/20	T: 5.59 ± 12.40 C: 68.10 ± 9.13	RM+ acupuncture	RM	20 min	Per day	14 days	① + ② + ③
Yu et al. (28)	33/33	T: 61.57 ± 4.73 C: 62.08 ± 5.11	RM+ acupuncture	RM	30 min	Per day	14 days	①
Tang et al. (43)	30/30	T: 52.12 ± 2.23 C: 52.48 ± 2.28	RM+ acupuncture	RM	30 min	Per day	14 days	① + ③
Deng et al. (29)	30/30	T: 63 ± 9.4 C: 61 ± 13.8	RM+ acupuncture	RM	30 min	Per day	42 days	① + ③
Wan et al. (30)	40/40	T: 61.2 ± 7.8 C: 60.4 ± 9.0	RM+ acupuncture	RM	30 min	Per day	30 days	① + ③
Du et al. (36)	28/29	T: 68.27 ± 11.86 C: 67.50 ± 9.74	RM+ acupuncture	RM	30 min	Per day	20 days	① + ② + ⑤
Zhang et al. (31)	24/26	T: 69.0 ± 9.89 C: 65.3 ± 10.51	RM+ acupuncture	RM	20 min	Per day	21 days	② + ③
Li et al. (32)	–	–	RM+ acupuncture	RM	30 min	Per day	30 days	① + ③

C, control; T, treatment; RM, routine medicine. ①, Fugl-Meyer Assessment (FMA); ②, NIH Stroke Scale (NIHSS); ③, Barthel Index (BI); ④, Modified Barthel Index (MBI); ⑤, Activities of Daily Living (ADL).

### 3.2 Risk of bias

**Figure 2:**
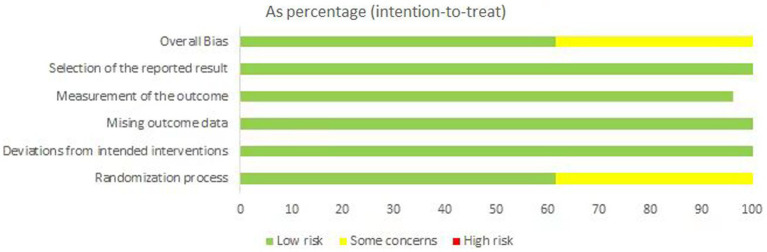
Risks of bias graph.

Quality assessment was performed for each included study. We used the revised Cochrane risk-of-bias tool for randomized trials (RoB2) to evaluate the bias risk. We assessed 16 studies as having “low risk of bias”. But considering undisclosed allocation concealment during the randomization process and ambiguous blinding assessment, 10 RCTs were evaluated as having “some concerns”. All studies were regarded as having a “low risk of bias” in deviations from intended interventions, missing outcome data, measurement of the outcome, and selection of the reporting result. The risks of bias are shown in Figure 2.

### 3.3 Effectiveness

#### 3.3.1 Primary outcome: limb dysfunction

##### 3.3.1.1 FMA

**Figure 3:**
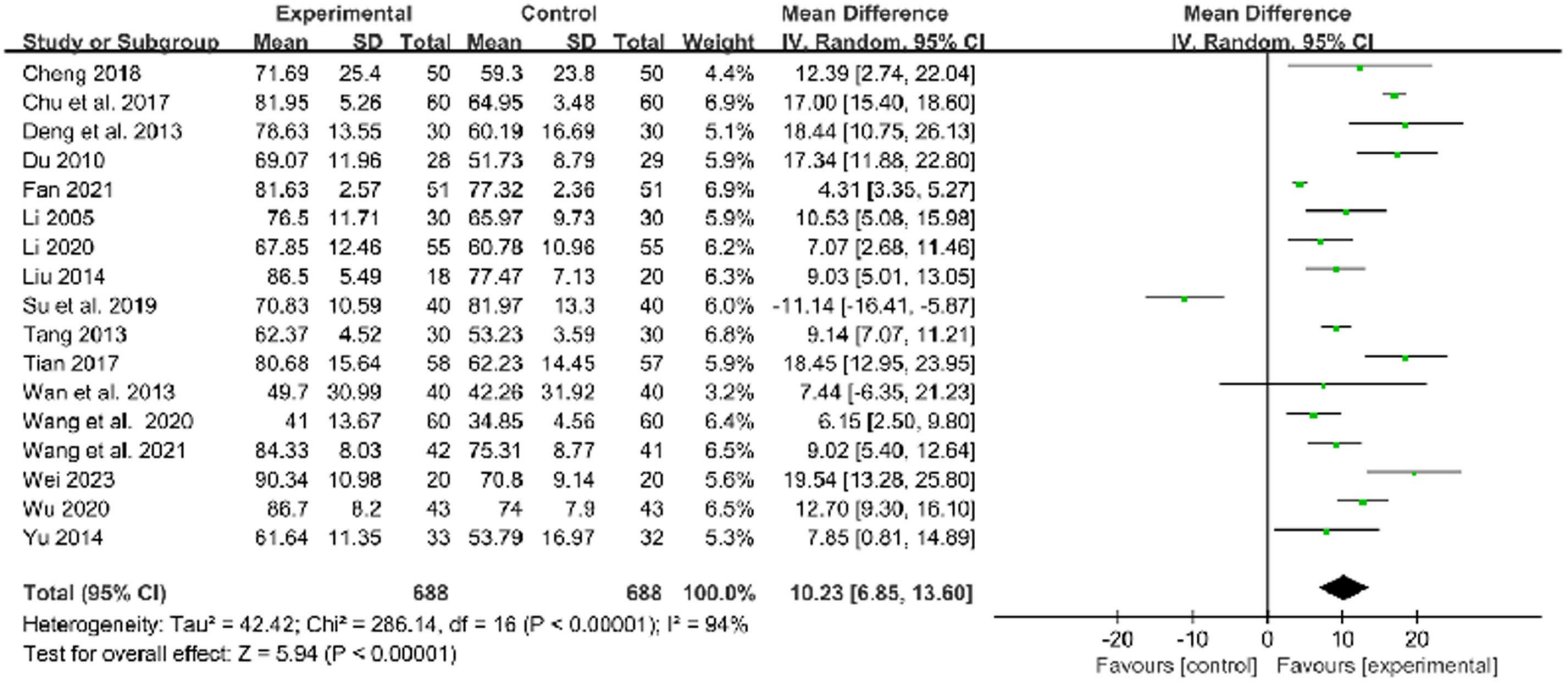
Forest plot of FMA comparison between combined therapy and RM group.

Seventeen studies used FMA to evaluate the limb dysfunction improvement and demonstrated that acupuncture and routine medicine (RM) combined therapy could be more effective than RM only (MD: 10.23, 95%CI: 6.85–13.60, *p* < 0.00001, heterogeneity (*p* < 0.00001), *I^2^* = 94%, Figure 3). The smallest treatment effect (10.23) exceeded the MCID (6).

**Figure 4:**
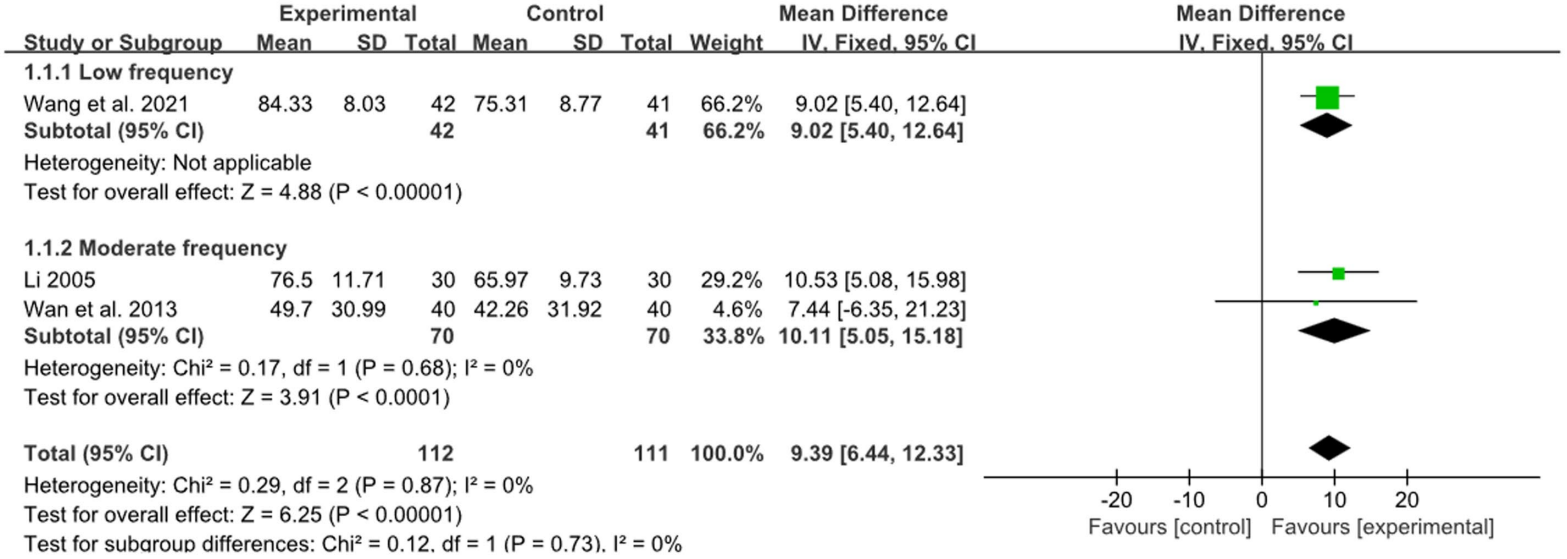
Effects of acupuncture on FMA based on different frequency.

One study reported the effect of low-frequency acupuncture on stroke, and 2 studies reported the effect of moderate-frequency acupuncture on stroke. The pooled results showed a pronounced superiority of combined therapy compared to RM for treatment of limb dysfunction within both the low frequency group and moderate frequency group (low frequency: MD: 9.02, 95%CI: 5.40–12.64, *p* < 0.00001, Figure 4; moderate frequency: MD: 10.11, 95%CI: 5.05–15.18, *p* < 0.00001, heterogeneity (*p* = 0.87), *I^2^* = 0%, Figure 4). The smallest treatment effect of the low frequency group (9.02) and moderate frequency group (10.11) exceeded the MCID (6).

**Figure 5:**
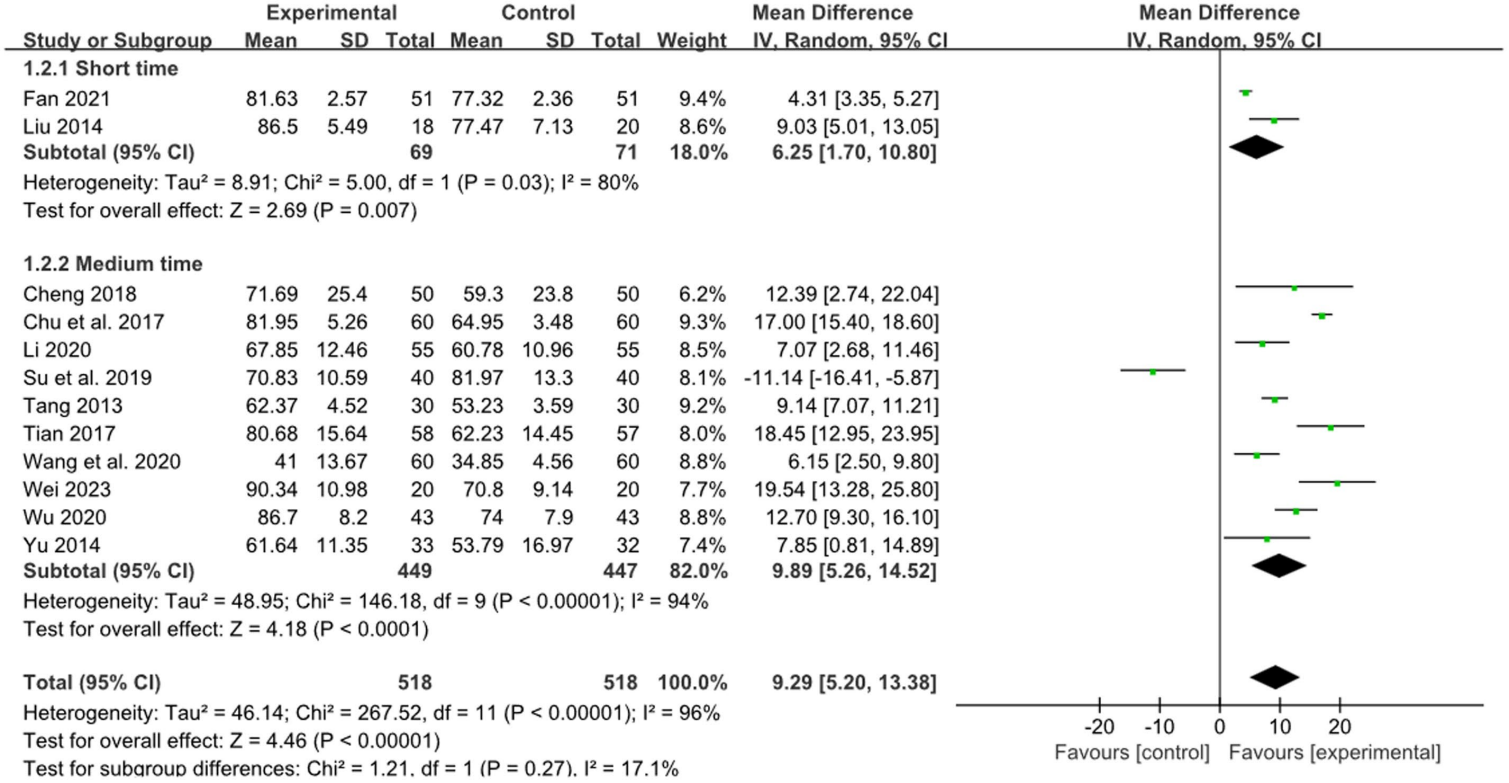
Effects of acupuncture on FMA based on different retention time.

Two studies reported the effect of short retention time on stroke, 10 studies reported the effect of medium retention time on stroke. The pooled results showed combined therapy was observed to have a significant difference compared to RM for treatment of limb dysfunction in the short and medium retention time group (short time: MD: 6.25, 95%CI: 1.70–10.80, *p* = 0.007, Figure 5; medium retention time: MD: 9.89, 95%CI: 5.26–14.52, *p* < 0.00001, heterogeneity (*p* = 0.03), *I^2^* = 94%, Figure 5). The smallest treatment effect of the short retention time group (6.25) and medium retention time (9.89) both exceeded the MCID (6).

**Figure 6:**
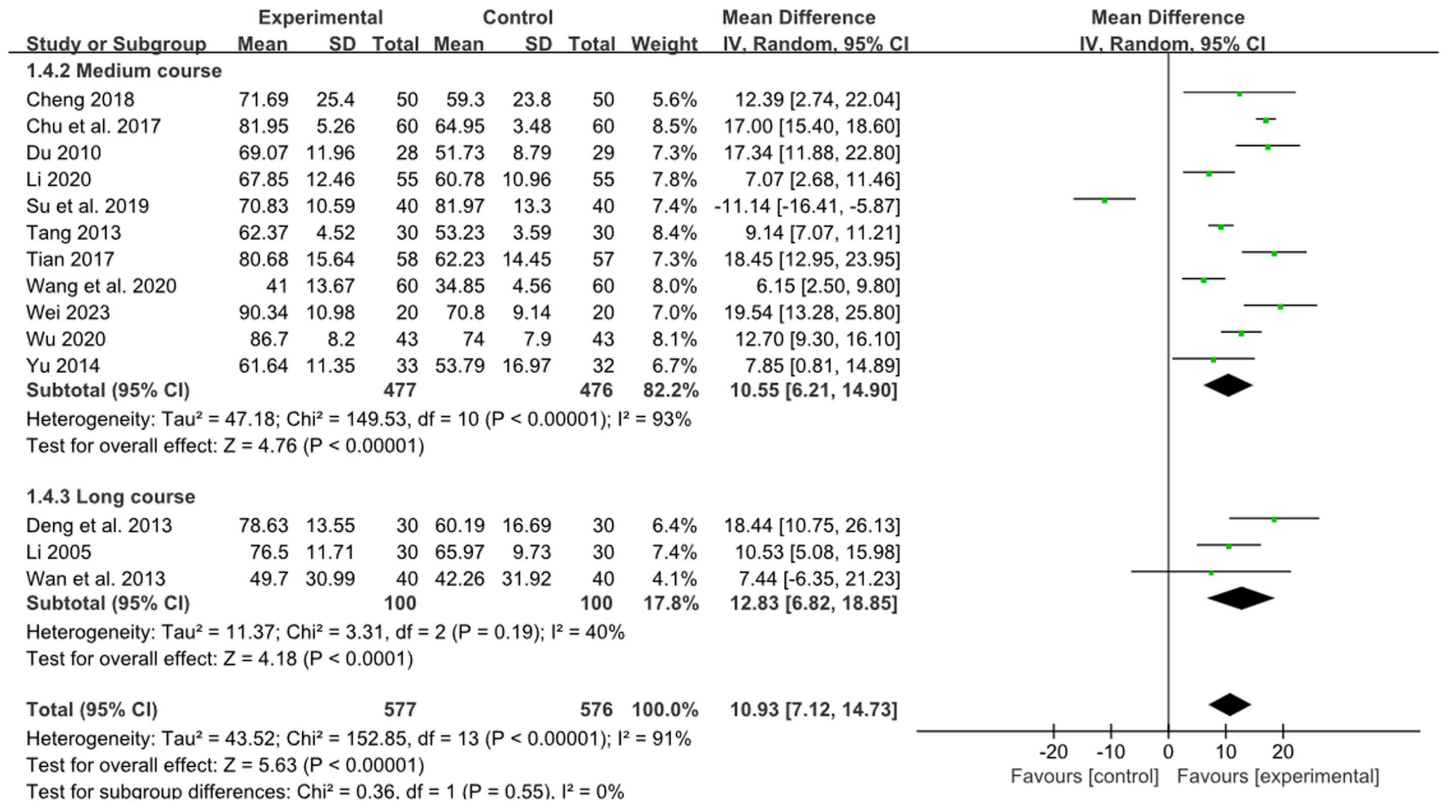
Effects of acupuncture on FMA based on different courses.

Eleven studies reported the effect of medium course acupuncture on stroke, 3 study reported the effect of long course acupuncture on stroke. The pooled results indicated a significantly greater effectiveness of combined therapy compared to RM in the treatment of limb dysfunction across the short and long course groups (medium course: MD: 10.55, 95%CI:6.21–14.90, *p* < 0.00001, heterogeneity (*p* < 0.00001), *I^2^* = 93%, Figure 6; long course: MD: 12.83, 95% CI: 6.82–18.85, *p* < 0.0001, heterogeneity (*p* = 0.19), *I^2^* = 40%, Figure 6). The smallest treatment effect of two kinds of course groups (10.55, 12.83) all exceeded the MCID (6).

The evidence concerning the effect of acupuncture with medium retention time and medium course on stroke was evaluated to be low according to GRADE due to the absence of blinded methods, limited employment of allocation hiding in most studies, and notable heterogeneity in the results, downgraded by risk of bias (−1 point) and inconsistency (−1 point). For effect of acupuncture with low frequency, moderate frequency, short time, and long course on stroke, we graded the evidence to be low quality according to GRADE due to the absence of blinded methods, limited employment of allocation hiding and small sample size in most studies, downgraded by risk of bias (−1 point) and imprecision (−1 point). The summary of findings is presented in the Supplementary material S1.

#### 3.3.2 Secondary outcome: neurological deficits and activities of daily living

##### 3.3.2.1 NIHSS

**Figure 7:**
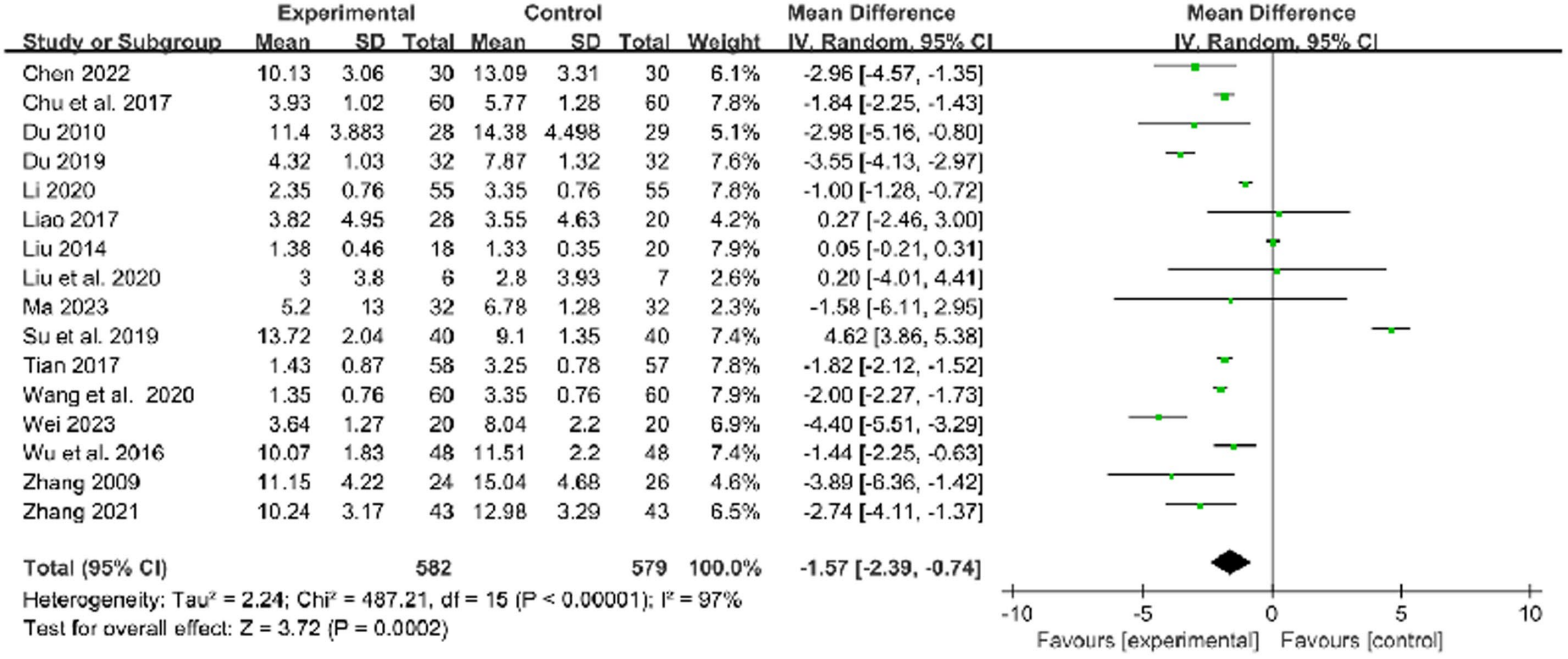
Forest plot of NIHSS comparison between combined therapy and RM group.

In 16 studies that used the NIHSS to assess treatment results, combined therapy appears to be more effective than RM in the treatment of limb dysfunction after stroke (MD: −1.57, 95% CI: −2.39 to −0.74, *p* = 0.0002, heterogeneity (*p* < 0.00001), *I^2^* = 97%, Figure 7).

One study reported the effect of high-frequency acupuncture on stroke. But in the high-frequency group, there was no difference between combined therapy and RM (high frequency: MD:0.20, 95% CI: −4.01 to 4.41, *p* = 0.93).

**Figure 8:**
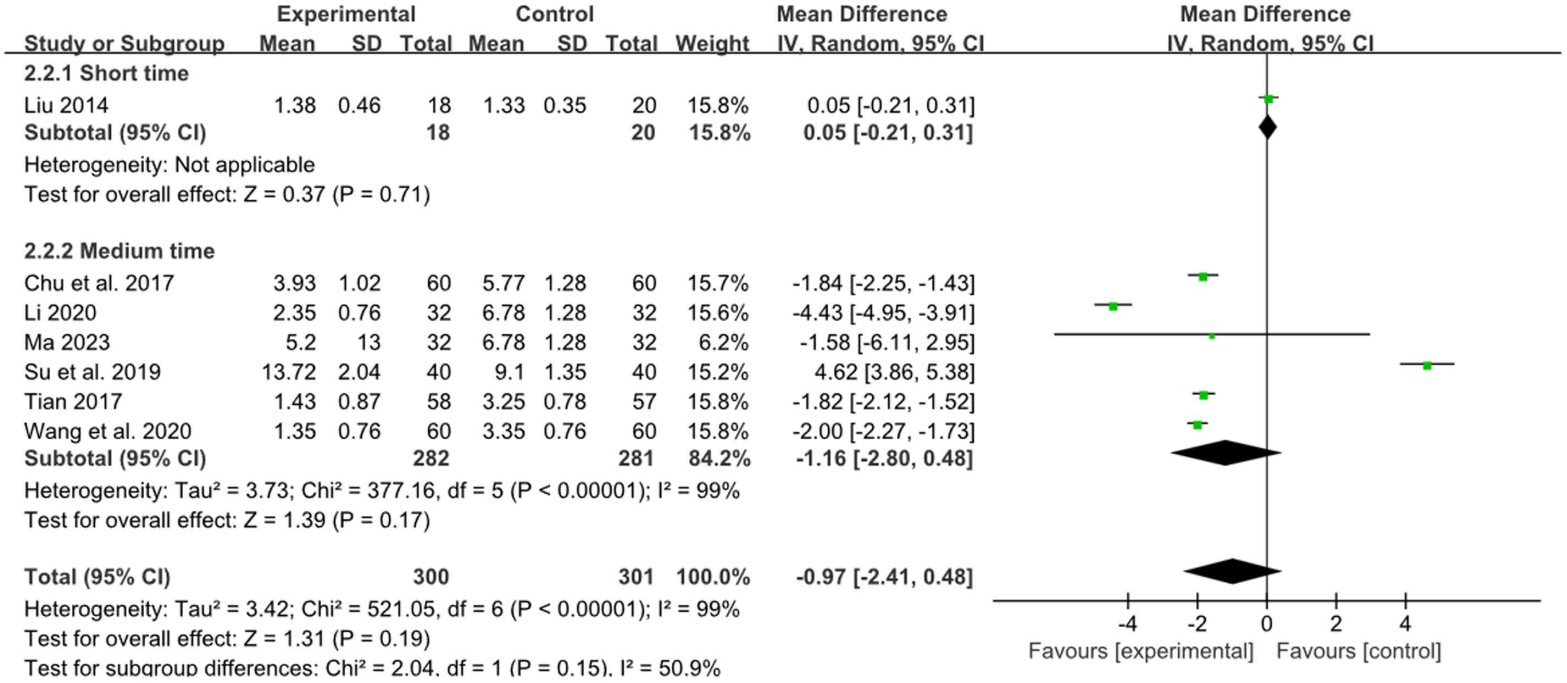
Effects of acupuncture on NIHSS based on different retention time.

One study reported the effect of short retention time on stroke, and 6 studies reported the effect of medium retention time on stroke. In the short and medium retention time group, there was no difference between combined therapy and RM (short retention time: MD: 0.05, 95% CI: −0.21–0.31, *p* = 0.71; medium retention time: MD: −1.16,95% CI: −2.80–0.48, *p* = 0.17, heterogeneity (*p* < 0.00001), *I^2^* = 99%, Figure 8).

**Figure 9:**
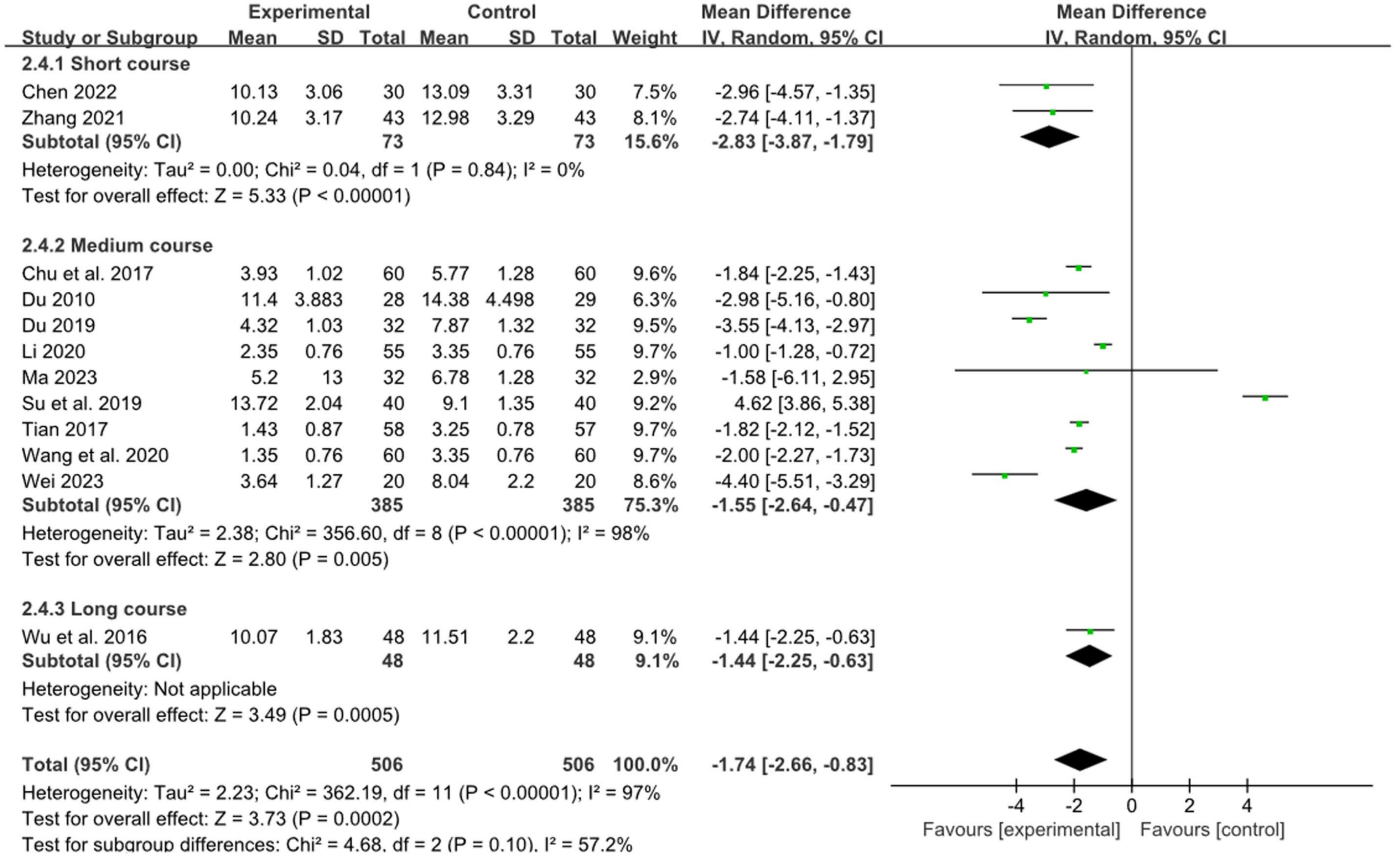
Effects of acupuncture on NIHSS based on different courses.

Two studies reported the effect of short course acupuncture on stroke, 9 studies reported the effect of medium course acupuncture on stroke, and one study reported the effect of long course acupuncture on stroke. In the short, medium, and long course group, the results show that combined therapy had a significantly greater effectiveness than RM for treatment of limb dysfunction (short course: MD: −2.83, 95% CI: −3.87 to −1.79, *p* < 0.00001, heterogeneity (*p* = 0.84), *I^2^* = 0%, Figure 9; medium course: MD: −1.55, 95% CI: −2.64 to −0.47, *p* = 0.005, heterogeneity (*p* < 0.00001), *I^2^* = 98%, Figure 9; long course: MD: −1.44, 95% CI: −2.25 to −0.63, *p* = 0.0005, Figure 9).

For the effect of acupuncture with medium retention time, and medium course on stroke, we graded the evidence to be low quality according to GRADE due to the absence of blinded methods, limited employment of allocation hiding in most studies, and notable heterogeneity in the results, downgraded by risk of bias (−1 point) and inconsistency (−1 point). For the effect of acupuncture with high frequency, short course, and long course on stroke, we graded the evidence to be low quality according to GRADE due to the absence of blinded methods, limited employment of allocation hiding and small sample size in most studies, downgraded by risk of bias (−1 point) and imprecision (−1 point). The summary of findings is presented in the Supplementary material S1.

##### 3.3.2.2 BI

**Figure 10:**
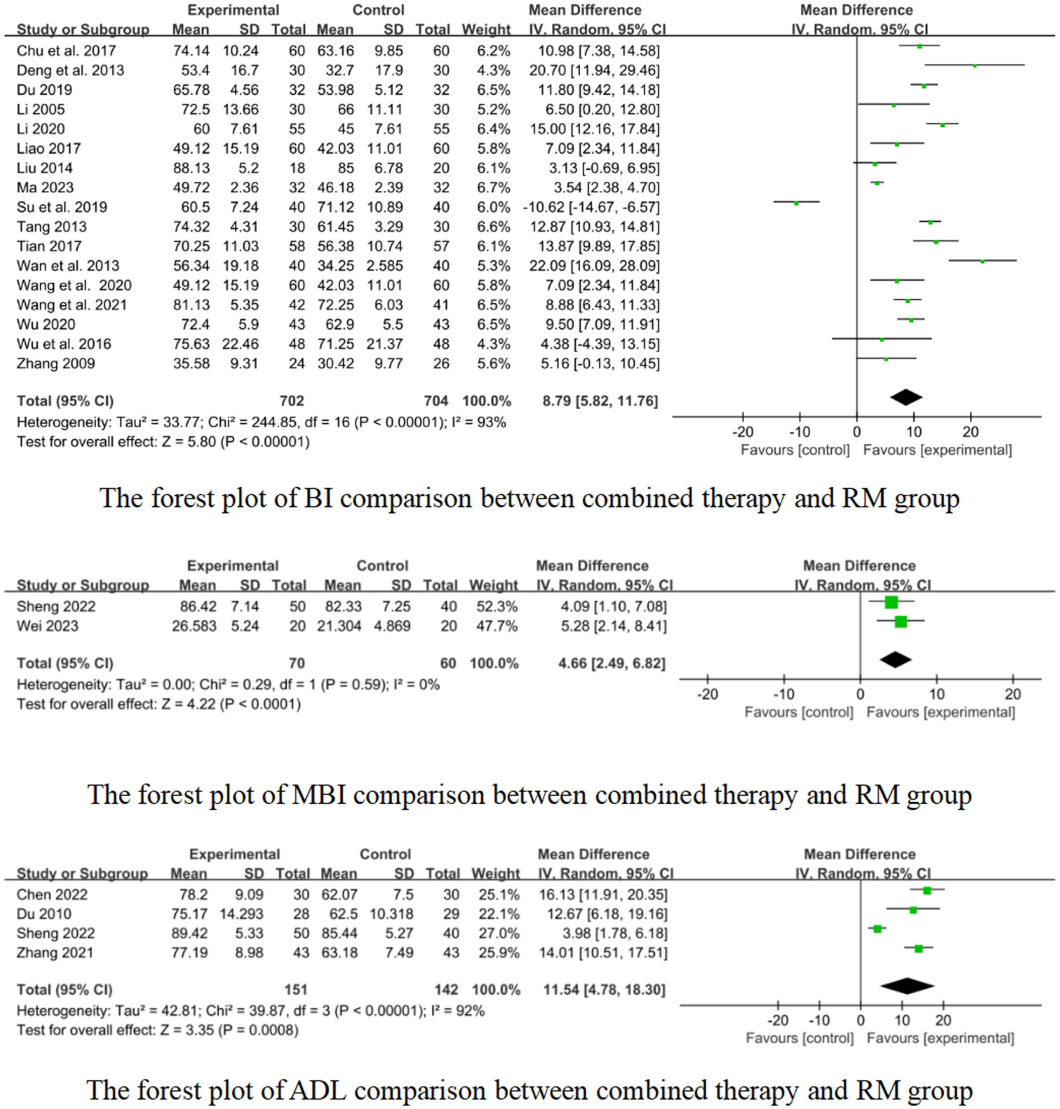
Forest plot of BI, MBI, and ADL comparison between combined therapy and RM group.

In 17 studies that used BI to assess treatment results, combined therapy appears to be more effective than RM for treatment of limb dysfunction after stroke (MD: 8.79, 95%CI: 5.82–11.76, *p* < 0.00001, heterogeneity (*p* < 0.00001), *I^2^* = 93%, Figure 10). The smallest treatment effect (8.79) exceeded the MCID (1.85).

**Figure 11:**
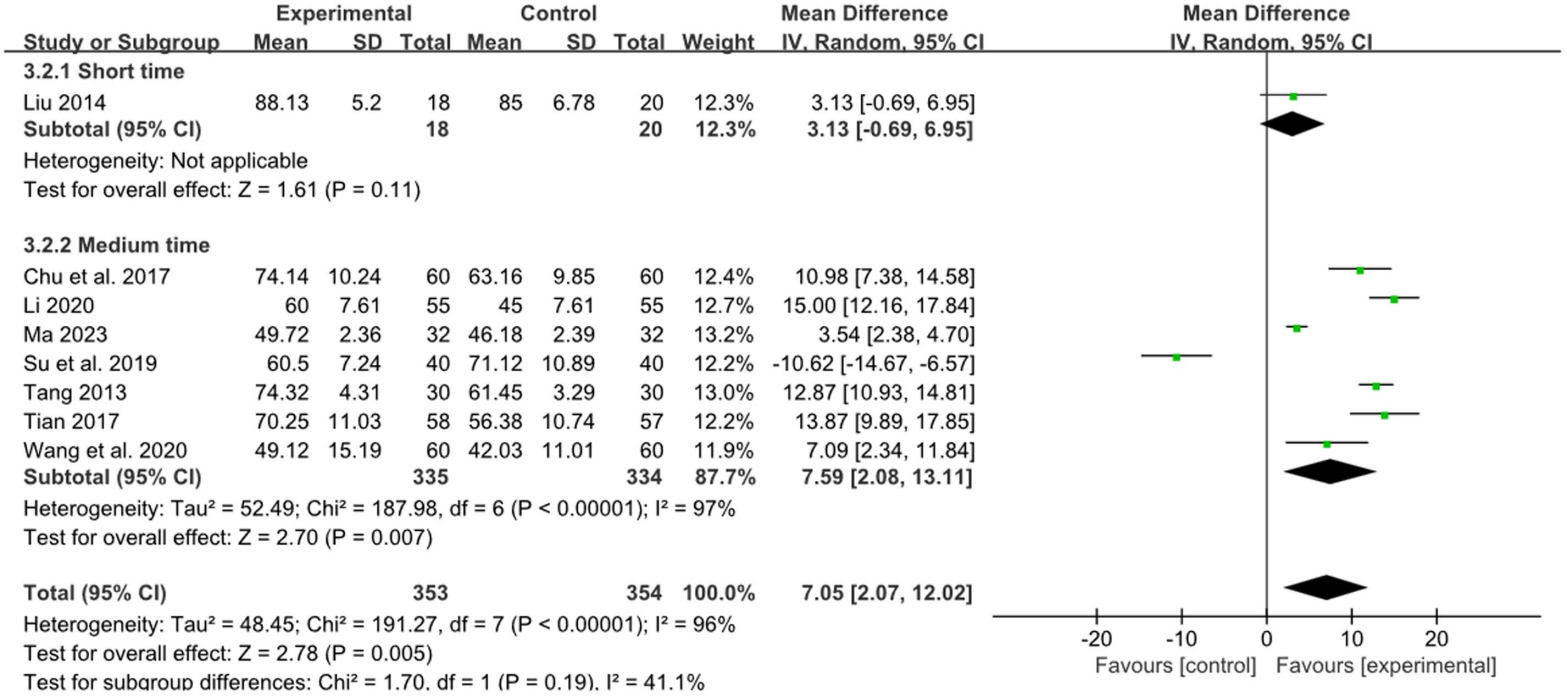
Effects of acupuncture on BI based on different retention time.

One study reported the effect of short retention time on stroke, and 7 studies reported the effect of medium retention time on stroke. The pooled results showed that there was a superior effect of combined therapy compared to RM for treatment of limb dysfunction in the medium retention time group (MD: 7.59, 95%CI: 2.08–13.11, *p* = 0.007, heterogeneity (*p* < 0.00001), *I^2^* = 97%, Figure 11). But in the short retention time group there was no difference between combined therapy and RM for treatment of limb dysfunction (MD: 3.13, 95% CI: −0.69–6.95, *p* = 0.11, Figure 11). The smallest treatment effect of the short retention time group (3.13) and medium retention time (7.59) both exceeded the MCID (1.85).

**Figure 12:**
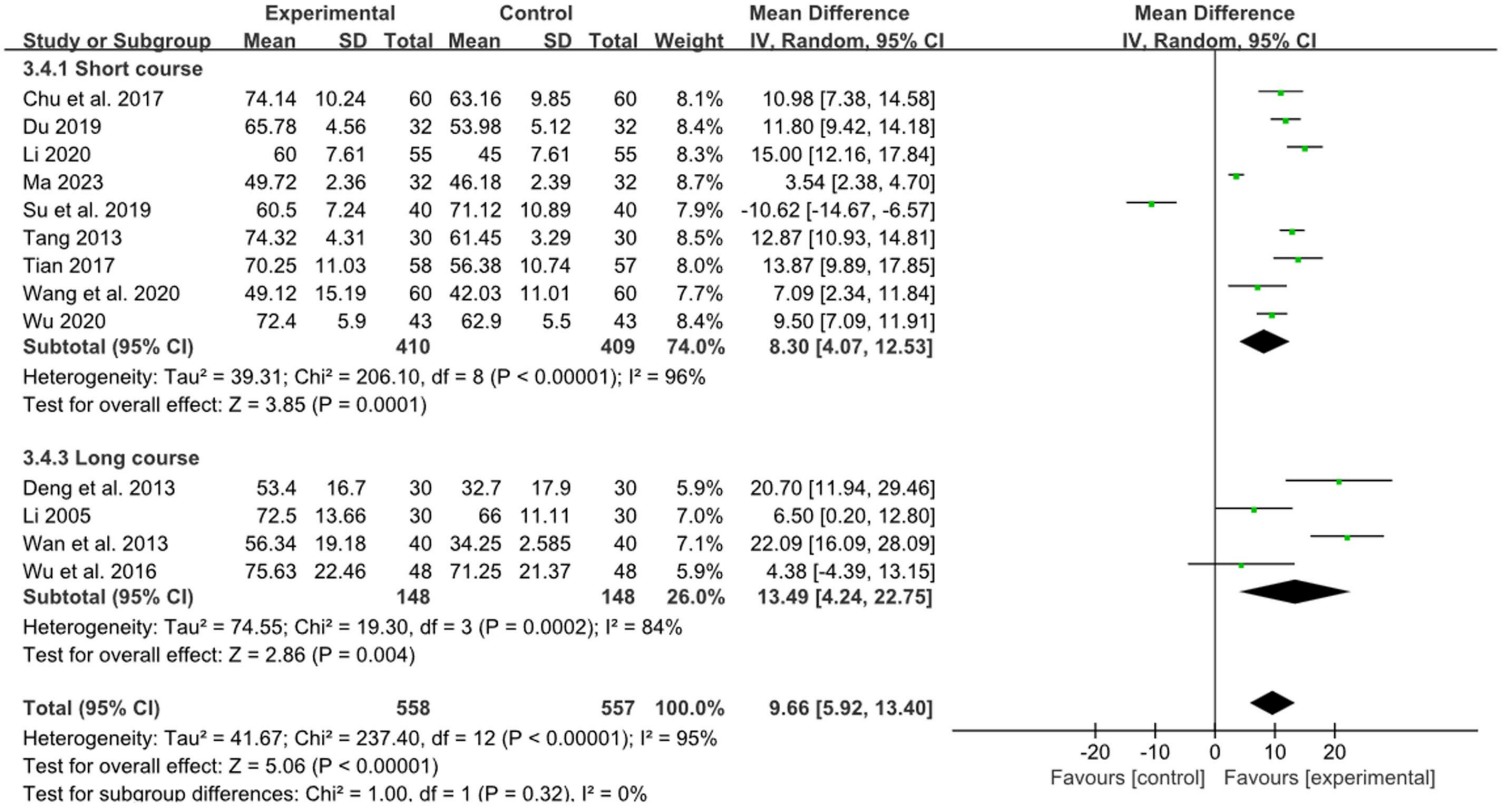
Effects of acupuncture on BI based on different courses.

Nine studies reported the effect of short course acupuncture on stroke, 4 studies reported the effect of long course acupuncture on stroke. In the short and long course group, the results show that combined therapy had a significantly greater effectiveness than RM for treatment of limb dysfunction (short course: MD:8.30, 95%CI: 4.07–12.53, *p* = 0.0001 heterogeneity (*p* < 0.00001), *I^2^* = 96% Figure 12; long course: MD:13.49, 95% CI: 4.24–22.75, *p* = 0.004, heterogeneity (*p* = 0.0002), *I^2^* = 84% Figure 12). The smallest treatment effect of the short course group (8.30) and long course (13.49) both exceeded the MCID (1.85).

For effect of acupuncture with medium retention time, short course, and long course on stroke, we graded the evidence to be low quality according to GRADE due to the absence of blinded methods, limited employment of allocation hiding in most studies, and notable heterogeneity in the results, downgraded by risk of bias (−1 point) and inconsistency (−1 point). For effect of acupuncture with short retention time, we graded the evidence to be low quality according to GRADE due to the absence of blinded methods, limited employment of allocation hiding and small sample size in most studies, downgraded by risk of bias (−1 point) and imprecision (−1 point). The summary of findings is presented in the Supplementary material S1.

##### 3.3.2.3 MBI

In 2 studies that used the MBI to assess treatment results, combined therapy could be more effective than RM for treatment of limb dysfunction after stroke (MD:4.66, 95% CI:2.49 to 6.82, *p* < 0.0001, heterogeneity (*p* = 0.59), *I^2^* = 0% Figure 10). The smallest treatment effect (4.66) fail to reach the MCID (5.34).

Two studies reported the effect of medium-course acupuncture on stroke. The results show that combined therapy had a significantly greater effectiveness than RM for treatment of limb dysfunction (MD:4.66, 95% CI: 2.49–6.82, *p* < 0.0001, heterogeneity (*p* = 0.59), *I^2^* = 0% Figure 10).

We graded the evidence to be very low quality according to GRADE due to the absence of blinded methods, limited employment of allocation hiding and small sample size in most studies, downgraded by risk of bias (−1 point) and imprecision (−2 point). The summary of findings is presented in the Supplementary material S1.

##### 3.3.2.4 ADL

In 4 studies that used the ADL to assess treatment results, combined therapy demonstrated potential superiority over RM in terms of effectiveness for treatment of limb dysfunction after stroke (MD:11.54, 95%CI:4.78 to 18.30, *p* = 0.0008, heterogeneity (*p* < 0.00001), *I^2^* = 92%, Figure 10). The smallest treatment effect (11.54) exceeded the MCID (5.9).

**Figure 13:**
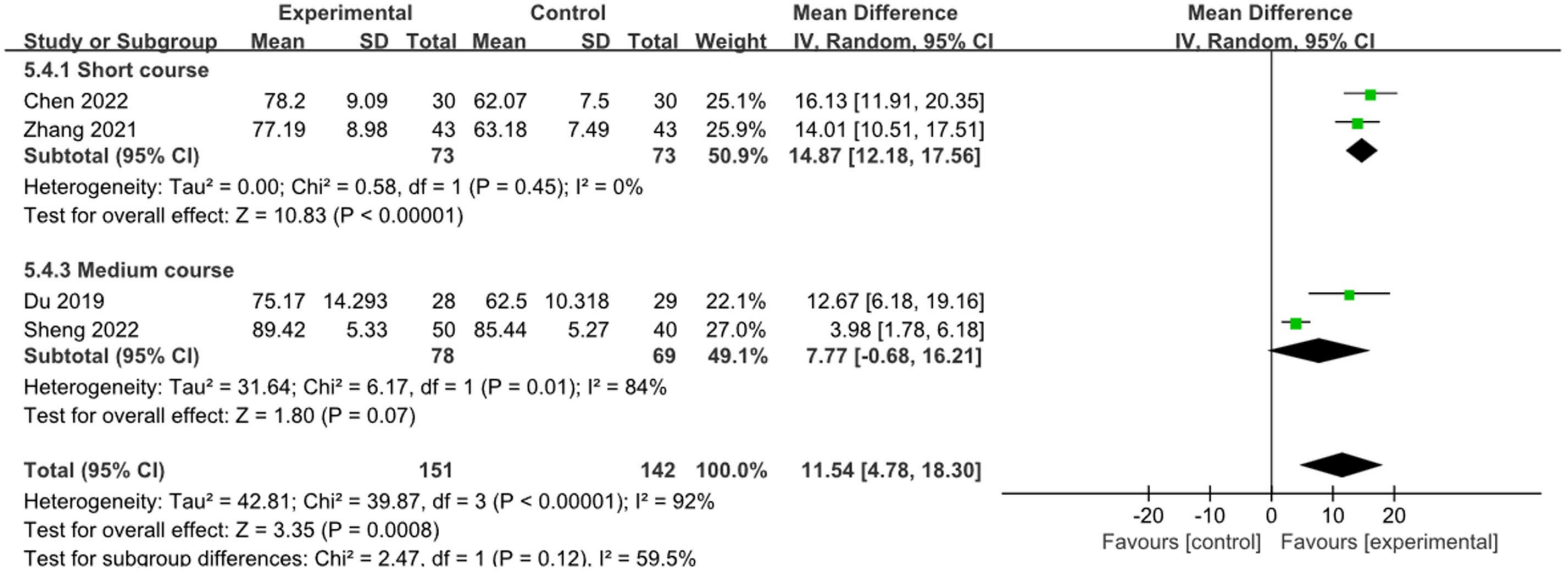
Effects of acupuncture on ADL based on different courses.

Two studies reported the effect of short course acupuncture on stroke, 2 studies reported the effect of medium course acupuncture on stroke. In the short course group, the results show that the combined therapy was significantly more effective than RM for treatment of limb dysfunction (MD: 14.87, 95% CI: 12.18–17.56, *p* < 0.00001, heterogeneity (*p* = 0.45), *I^2^* = 0%, Figure 13). But in the medium course group, there was no difference between combined therapy and RM (MD: 7.77, 95% CI: −0.68–16.21, *p* = 0.07, heterogeneity (*p* = 0.01), *I*^2^ = 84%, Figure 13). The smallest treatment effect of the short course group (14.87) and medium course (7.77) both exceeded the MCID (5.9).

For the effect of acupuncture with medium course on stroke, we graded the evidence to be low quality according to GRADE due to the absence of blinded methods, limited employment of allocation hiding in most studies, notable heterogeneity in the results, and small sample size, downgraded by risk of bias (−1 point), inconsistency (−1 point) and imprecision (−1 point). For the effect of acupuncture with short course on stroke, we graded the evidence to be low quality according to GRADE due to the absence of blinded methods, limited employment of allocation hiding in most studies and small sample size, downgraded by risk of bias (−1 point) and imprecision (−1 point). The summary of findings is presented in the Supplementary material S1.

### 3.4 Sensitivity analyses

For the FMA, BI, NIHSS, and ADL, sensitivity analyses showed that the results remained essentially unchanged after omitting any one study. It suggests that the research results are credible and relatively stable. The results of sensitivity analyses were shown in the Supplementary material S2.

### 3.5 Assessment of publication bias

The funnel plot of FMA, BI, MBI, NIHSS, and ADL between different groups showed symmetry, so incorporating research showed no significant publication bias (see Supplementary material S2). Egger’s test of FMA (*p* = 0.265), BI (*p* = 0.363), NIHSS (*p* = 0.702), and ADL (*p* = 0.213) showed no publication bias. We did not perform Egger’s test for MBI because of the small sample size.

## 4 Discussion

### 4.1 Summary of the main findings

By comprehensively searching relevant studies, 26 RCTs with 1947 participants were included in this review. Our meta-analysis showed low (every other day) and moderate frequency (once a day), short course treatment (<2 weeks) are effective in improving limb dysfunction in acute ischemic stroke. We did not find the optimal intervention dose for needle retention time.

### 4.2 Comparison with existing studies

To the best of our knowledge, this is the first systematic review and meta-analysis that comprehensively summarized evidence regarding the dose-related efficacy of acupuncture for limb dysfunction after acute stroke.

In terms of interval time, this review compares the effect of different acupuncture intervals time on limb dysfunction after stroke. The results showed that low-frequency (every other day) and moderate-frequency (once a day) is beneficial for the recovery of limb dysfunction, and high-frequency (twice a day) was not statistically significant. Current research indicates that the cumulative effect of acupuncture does not correspond linearly to the increase in the frequency of acupuncture. Rather, it exhibits a specific pattern of waxing and waning (47). Once a certain threshold is surpassed, the body’s responsiveness diminishes, and the acupuncture effect may plateau or decline slightly (48). The high-frequency may make the body tolerate it and affect the therapeutic effect of acupuncture. But previous RCTs have shown that the effect of high-frequency (twice a day) is better than moderate-frequency (once a day) in treating hemiplegia after stroke (49–52). This differs from our results, and more high-quality RCTs will be needed to verify this in the future.

We did not obtain sufficient evidence regarding the optimal intervention dose for acupuncture retention time. The current research mainly focuses on the medium and short retention time. The number of studies with long retention time and sample size were small. The ancient Chinese medicine book “Ling Shu-Nine Needle and Twelve Original” highlights the potential risks associated with improper needle retention times. It states, “if the needle is retained in the acupoint without withdrawal, it can lead to leakage of vital essence; whereas if the needle is withdrawn from the acupoint too fast, it can cause the lingering of harmful substances. Leakage of vital essence can worsen the disease and result in a weakened constitution, while the lingering of harmful substances can manifest as carbuncles and ulcers.” Previous research has shown that medium retention time is considered the optimal induction period for acupuncture, as prolonged application beyond this duration has shown a decline in its effectiveness (53). However, other study has found that the long retention time may be associated with better efficacy of acupuncture and prolonged retention of needles can accumulate curative effect and promote the recovery of limb function (54). In the future, more studies are needed to confirm the relationship between acupuncture retention time and efficacy.

Regarding course, our review showed that the effect of acupuncture with a course of short course may be beneficial. It is related to the “after-effect” of acupuncture therapy, which means the effects of acupuncture can last for some time after stopping treatment (55). Continuous acupuncture may lead to “fatigue” of acupuncture points, which is difficult to induce menstrual qi and greatly reduces the efficacy of acupuncture (56). Studies have shown that for the treatment of limb dysfunction in acute ischemic stroke, the short course of acupuncture can promote the recovery of limb motor function, neurological function impairment and daily living ability (57, 58). Moreover, acupuncture for a long time is easy to increase the pain of patients and the economic burden of families. As a result, we can choose a short course of acupuncture for patients with post-stroke limb dysfunction that requires prolonged acupuncture rehabilitation.

### 4.3 Limitation

There are several limitations in this systematic review. Firstly, we did not strictly limit the methods of acupuncture (such as auricular acupuncture, head acupuncture), and different acupuncture methods may have some impact on the results. Secondly, patients included in the trials had different initial limb dysfunction levels, resulting in possible clinical heterogeneity between studies. Thirdly, we only included studies in Chinese and English, other language studies may be missed.

## 5 Conclusion

After three-step analysis, we concluded that acupuncture is an effective treatment capable of improving limb dysfunction after acute stroke. A dose-effect relationship was found between the dose (frequency, needle retention time and course) of acupuncture and clinical response. Comparing several different doses will help to explore the best treatment options and guide clinical decision making. However, further studies are needed to confirm the dose-effect relationship of acupuncture in the treatment of this disorder.

## Data availability statement

The original contributions presented in the study are included in the article/Supplementary material, further inquiries can be directed to the corresponding author.

## Author contributions

XW: Conceptualization, Software, Writing – original draft, Data curation. LuX: Formal analysis, Methodology, Software, Writing – original draft. LiX: Conceptualization, Supervision, Writing – review & editing. CT: Investigation, Writing – review & editing. YL: Investigation, Writing – review & editing. XD: Supervision, Writing – review & editing.
