# Supplementary material for: The dose-effect relationship of acupuncture on limb dysfunction after acute stroke: a systematic review and meta-analysis
Source: Front Neurol. 2024 Feb 28;15:1341560. doi: 10.3389/fneur.2024.1341560 (PMC10933065; doi:10.3389/fneur.2024.1341560)
Supplement: Supplementary file 1 [file Data_Sheet_1.PDF]

Author(s):
Question: Acupuncture compared to RM for limb dysfunction after acute stroke
Setting:
Bibliography:

Certainty assessment							№ of patients		Effect		Certainty	Importance
№ of studies	Study design	Risk of bias	Inconsistency	Indirectness	Imprecision	Other considerations	Acupuncture	RM	Relative (95% CI)	Absolute (95% CI)		
**FMA**												
17	randomised trials	serious[a,b]	serious[c]	not serious	not serious	none	688	688	-	MD **10.23 higher** (6.85 higher to 13.6 higher)	⨁⨁◯◯ Low	
**Low frequency**												
1	randomised trials	serious[a,b,d]	not serious	not serious	serious[d]	none	42	41	-	MD **9.02 higher** (5.4 higher to 12.64 higher)	⨁⨁◯◯ Low	
**Moderate frequency**												
2	randomised trials	serious[a,b]	serious[c]	not serious	not serious	none	646	647	-	MD **10.11 higher** (5.05 higher to 15.18 higher)	⨁⨁◯◯ Low	
**Short time**												
2	randomised trials	serious[a,b]	not serious	not serious	serious[d]	none	69	71	-	MD **6.25 higher** (1.7 higher to 10.8 higher)	⨁⨁◯◯ Low	
**Medium time**												
10	randomised trials	serious[a,b]	serious[c]	not serious	not serious	none	619	617	-	MD **9.89 higher** (5.26 higher to 14.52 higher)	⨁⨁◯◯ Low	
**Medium course**												
11	randomised trials	serious[a,b]	not serious	not serious	serious[d]	none	28	29	-	MD **10.55 higher** (6.21 higher to 14.9 higher)	⨁⨁◯◯ Low	
**Long course**												
3	randomised trials	serious[a,b]	not serious	not serious	serious[d]	none	263	262	-	MD **12.83 higher** (6.82 higher to 18.85 higher)	⨁⨁◯◯ Low	
**NIHSS**												
16	randomised trials	serious[a,b]	serious[c]	not serious	not serious	none	582	579	-	MD **1.57 lower** (2.39 lower to 0.74 lower)	⨁⨁◯◯ Low	
**High frequency**												
1	randomised trials	serious[a,b]	not serious	not serious	serious[d]	none	6	7	-	MD **0.2 higher** (4.01 lower to 4.41 higher)	⨁⨁◯◯ Low	
**Short time**												
1	randomised trials	serious[a,b]	not serious	not serious	serious[d]	none	70	66	-	MD **0.05 higher** (0.21 lower to 0.31 higher)	⨁⨁◯◯ Low	
**Medium time**												

6	randomised trials	serious[a,b]	serious[c]	not serious	not serious	none	512	513	-	MD **1.16 lower** (2.8 lower to 0.48 higher)	⊕⊕◯◯ Low	
**Short course**												
2	randomised trials	serious[a,b]	not serious	not serious	serious[d]	none	402	404	-	MD **2.83 lower** (3.87 lower to 1.79 lower)	⊕⊕◯◯ Low	
**Moderate course**												
9	randomised trials	serious[a,b]	serious[c]	not serious	not serious	none	52	55	-	MD **1.55 lower** (2.64 lower to 0.47 lower)	⊕⊕◯◯ Low	
**Long course**												
1	randomised trials	serious[a,b]	not serious	not serious	serious[d]	none	128	120	-	MD **1.44 lower** (2.25 lower to 0.63 lower)	⊕⊕◯◯ Low	
**BI**												
17	randomised trials	serious[a,b]	serious[c]	not serious	not serious	none	702	704	-	MD **8.79 higher** (5.82 higher to 11.76 higher)	⊕⊕◯◯ Low	
**Short time**												
1	randomised trials	serious[a,b]	not serious	not serious	serious[d]	none	24	26	-	MD **3.13 higher** (0.69 lower to 6.95 higher)	⊕⊕◯◯ Low	
**Medium time**												
7	randomised trials	serious[a,b]	serious[c]	not serious	not serious	none	600	598	-	MD **7.59 higher** (2.08 higher to 13.11 higher)	⊕⊕◯◯ Low	
**Short course**												
9	randomised trials	serious[a,b]	serious[c]	not serious	not serious	none	396	397	-	MD **8.3 higher** (4.07 higher to 12.53 higher)	⊕⊕◯◯ Low	
**Long course**												
4	randomised trials	serious[a,b]	serious[c]	not serious	not serious	none	282	281	-	MD **13.49 higher** (4.24 higher to 22.75 higher)	⊕⊕◯◯ Low	
**MBI**												
2	randomised trials	serious[a,b]	not serious	not serious	very serious[d]	none	70	60	-	MD **4.66 higher** (2.49 higher to 6.82 higher)	⊕◯◯◯ Very low	
**ADL**												
4	randomised trials	serious[a,b]	serious[c]	not serious	serious[d]	none	151	142	-	MD **11.54 higher** (4.78 higher to 18.3 higher)	⊕◯◯◯ Very low	
**Short course**												
2	randomised trials	serious[a,b]	serious[c]	not serious	serious[d]	none	123	113	-	MD **14.87 higher** (12.18 higher to 17.56 higher)	⊕◯◯◯ Very low	

**Medium course**												
2	randomised trials	serious[a,b]	not serious	not serious	serious[d]	none	28	29	-	MD **7.77 higher** (0.68 lower to 16.21 higher)	⨁⨁◯◯ Low	

CI: confidence interval; **MD:** mean difference

Explanations

a. Blind methods were not used in the study.
b. Study not all use allocation hiding.
c. There was heterogeneity in the results.
d. The sample size is small.
