# Supplementary figures and images for: The dose-effect relationship of acupuncture on limb dysfunction after acute stroke: a systematic review and meta-analysis
Source: Front Neurol. 2024 Feb 28;15:1341560. doi: 10.3389/fneur.2024.1341560 (PMC10933065; doi:10.3389/fneur.2024.1341560)

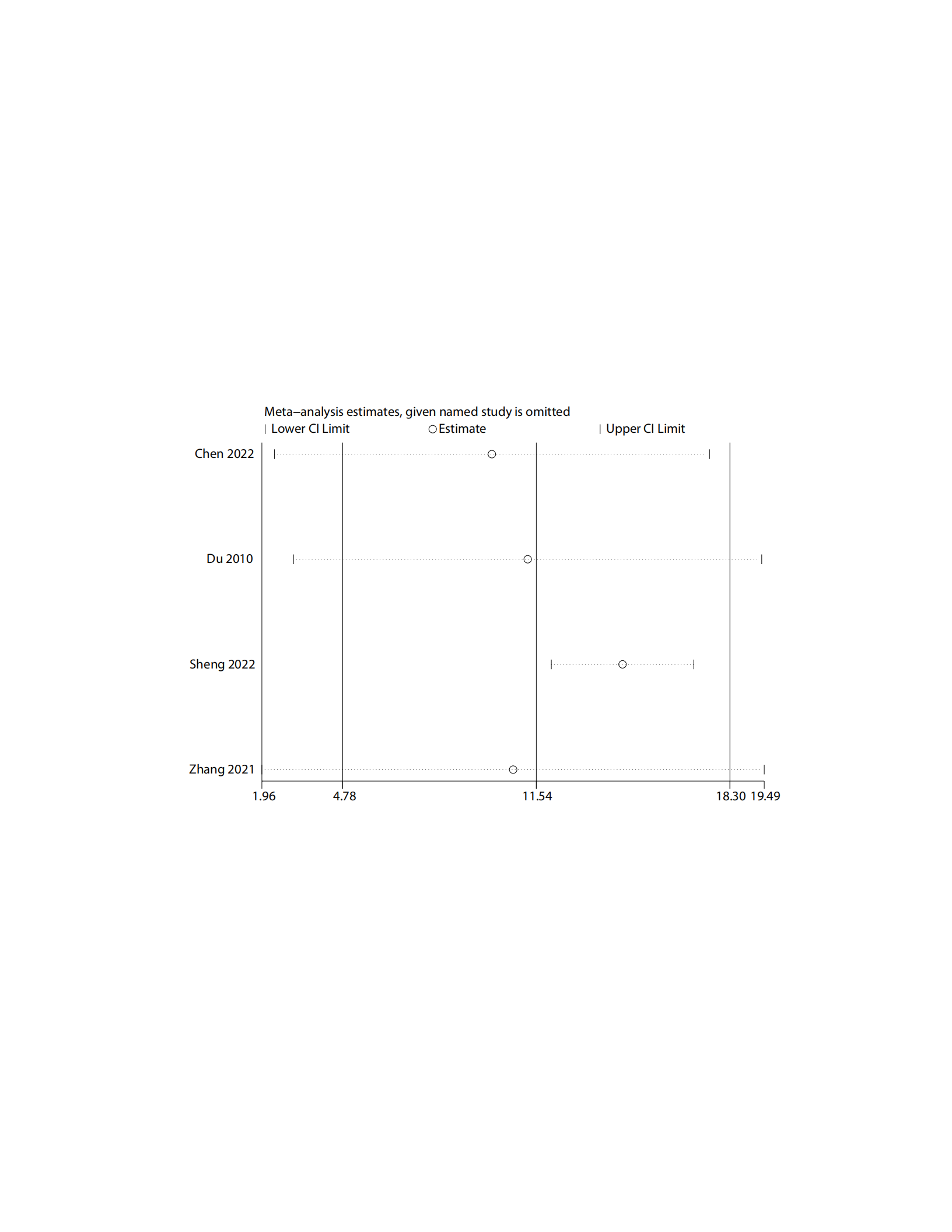

Supplement: Supplementary file 2 [file Data_Sheet_2.ZIP › supplementary data-sensitivity analyses/Sensitivity analysis of ADL omitting single studies in overall studies..tif]

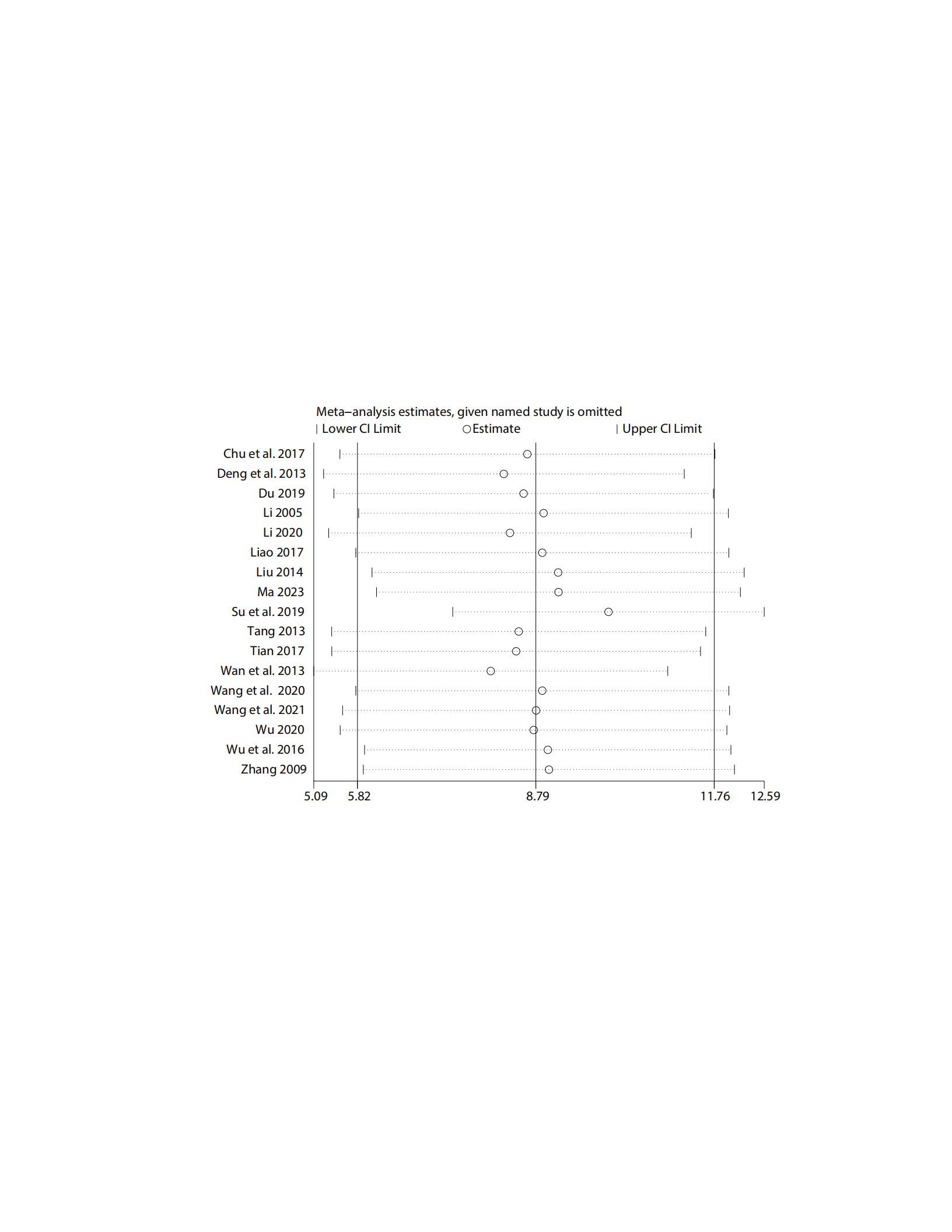

Supplement: Supplementary file 2 [file Data_Sheet_2.ZIP › supplementary data-sensitivity analyses/Sensitivity analysis of BI omitting single studies in overall studies..tif]

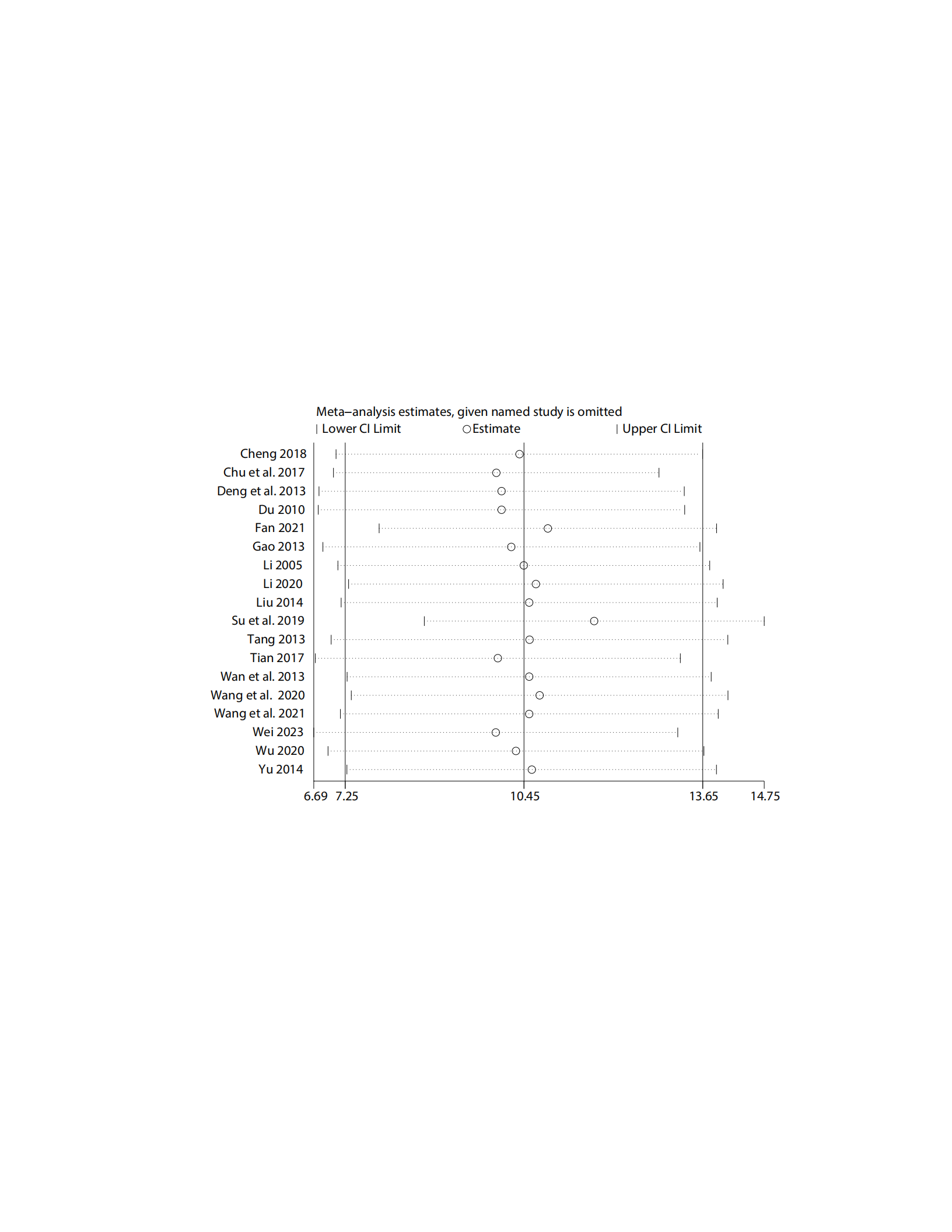

Supplement: Supplementary file 2 [file Data_Sheet_2.ZIP › supplementary data-sensitivity analyses/Sensitivity analysis of FMA omitting single studies in overall studies..tif]

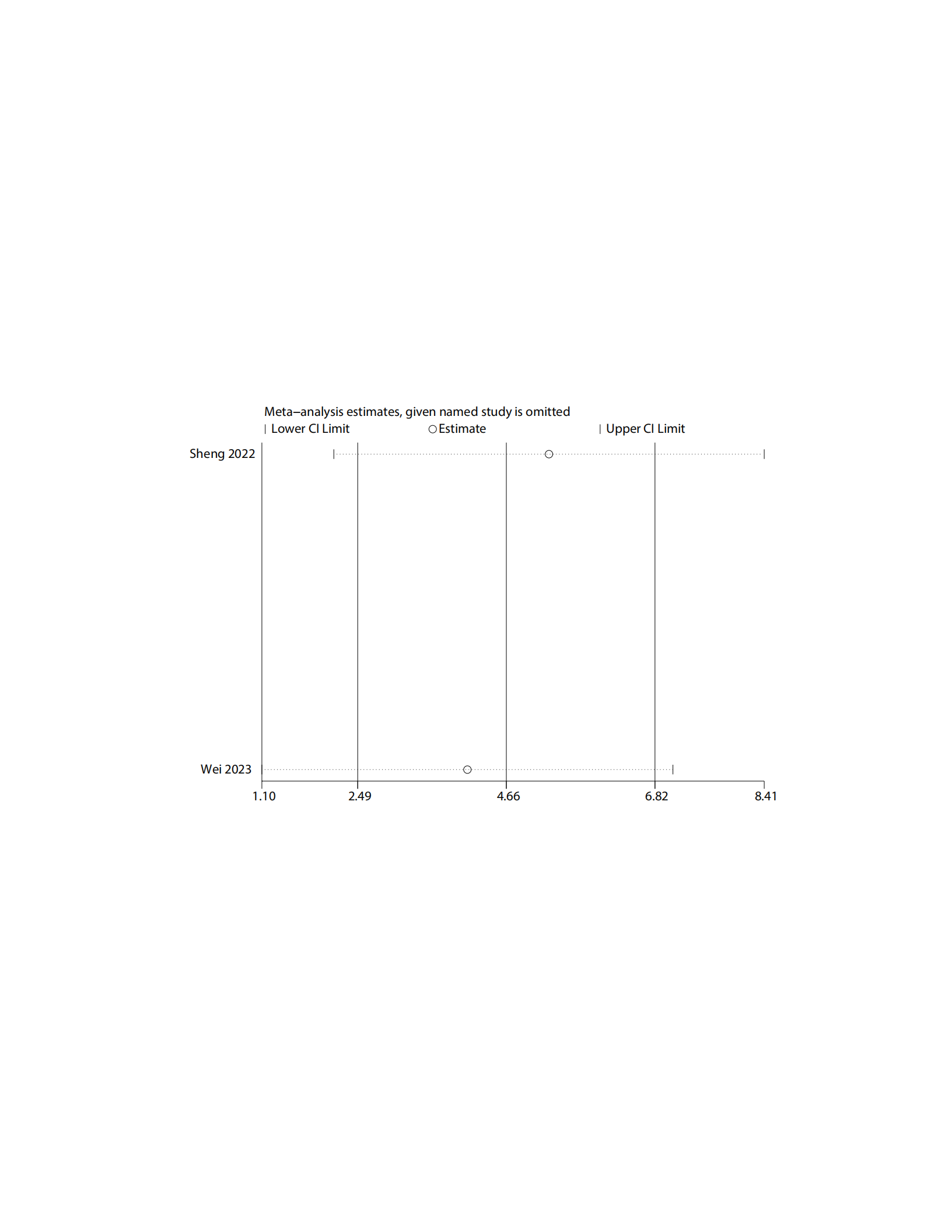

Supplement: Supplementary file 2 [file Data_Sheet_2.ZIP › supplementary data-sensitivity analyses/Sensitivity analysis of MBI omitting single studies in overall studies..tif]

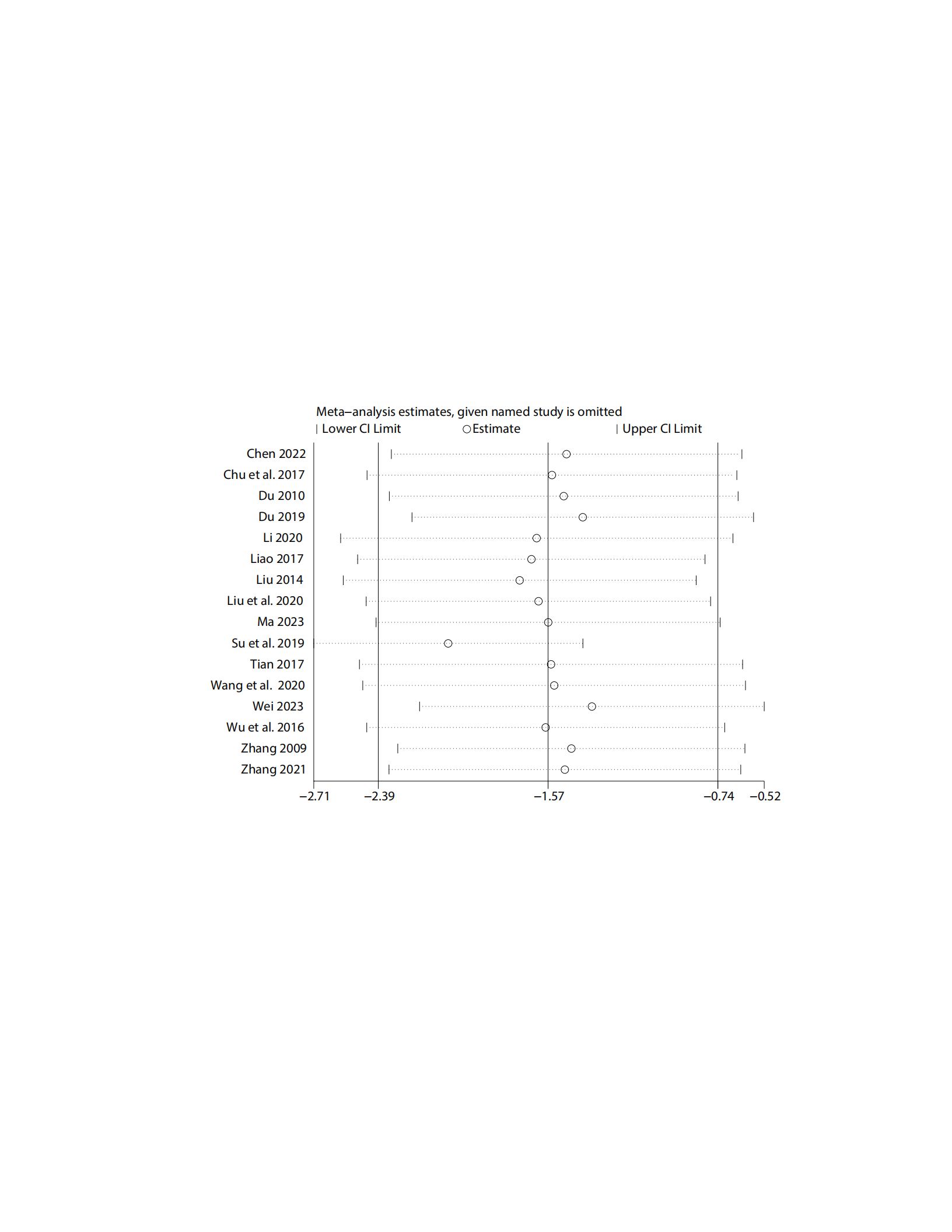

Supplement: Supplementary file 2 [file Data_Sheet_2.ZIP › supplementary data-sensitivity analyses/Sensitivity analysis of NIHSS omitting single studies in overall studies..tif]

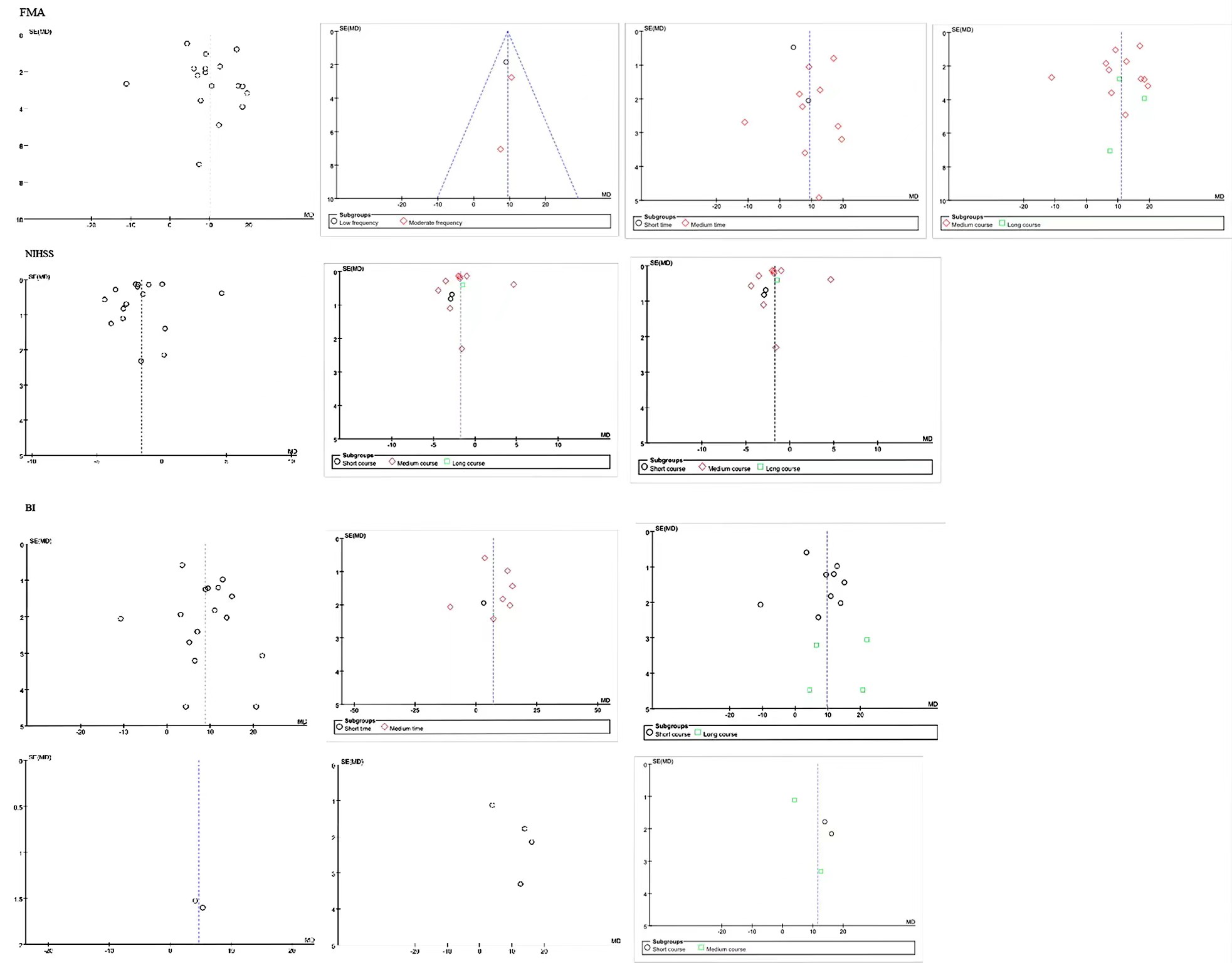

Supplement: Supplementary file 2 [file Data_Sheet_2.ZIP › Funnel plot.jpg]
